# Fecal microbiota transplantation from patients with autoimmune encephalitis modulates Th17 response and relevant behaviors in mice

**DOI:** 10.1038/s41420-020-00309-8

**Published:** 2020-08-11

**Authors:** Hao Chen, Zhaoyu Chen, Liping Shen, Xiuhua Wu, Xueying Ma, Dengna Lin, Man Zhang, Xiaomeng Ma, Yingying Liu, Zhanhang Wang, Yuefeng Zhang, Zuying Kuang, Zhiwei Lu, Xuefei Li, Lili Ma, Xiuli Lin, Lei Si, Xiaohong Chen

**Affiliations:** 1 Department of Neurology and Multiple Sclerosis Research Center, The Third Affiliated Hospital, Sun Yat-Sen University, 600 Tianhe Road, 510630 Guangzhou, Guangdong Province China; 2 Department of Psychiatry, Third Affiliated Hospital, Sun Yat-Sen University, Guangzhou, China; 3 Department of Infectious Diseases, Third Affiliated Hospital, 510630 Guangzhou, China; 4 School of Environmental and Biological Sciences, The State University of New Jersey, New Brunswick, NJ USA; 5 Department of Neurology, Guangdong 999 Brain Hospital, Guangzhou, China; 6 Department of Neurology, Affiliated Brain Hospital of Guangzhou Medical University, Guangzhou, China; 7 Yanke Biotechnology Co., Guangzhou, China

**Keywords:** Diseases, Immunology, Microbiology

## Abstract

The significance of the microbiota-gut-brain axis has been increasingly recognized as a major modulator of autoimmunity. Here, we aim to characterize the gut microbiota of a large cohort of treatment-naïve anti-*N*-methyl-d-aspartate receptor (anti-NMDAR) encephalitis patients relative to that of healthy controls (HCs). Relative to HCs, anti-NMDAR encephalitis patients had a decreased microbiome alpha-diversity index, marked disturbances of gut microbial composition and intestinal permeability damage. Disturbed microbiota in anti-NMDAR encephalitis patients might be linked with different clinical characteristics. Imputed KEGG analysis revealed perturbations of functional modules in the gut microbiomes of anti-NMDAR encephalitis. Compared to HCs, microbiota-depleted mice receiving fecal microbiota transplantation (FMT) from anti-NMDAR encephalitis patients had hypersensitivity and cognitive impairment. Furthermore, anti-NMDAR encephalitis FMT mice showed altered T cells in the spleen and small intestine lamina propria with an increased Th17 cells. Overall, this study first suggests that the anti-NMDAR encephalitis microbiome itself can influence neurologic, Th17 response and behavioral function. The gut microbiota is a potential therapeutic target for anti-NMDAR encephalitis.

## Introduction

Encephalitis, a neurological disorder caused by inflammation of the brain parenchyma, has an estimated incidence of 5–10 people per 100,000 people per year, although this incidence is likely an underestimation^1^. Although encephalitis is most commonly attributed to underlying viral infection, autoimmune conditions have become increasingly appreciated as causes of encephalitis^2^. Autoimmune encephalitis (AIE) is associated with different antibodies directed towards mainly synaptic receptors, including the *N*-methyl-d-aspartate receptor (NMDAR), the α-amino-3-hydroxy-5-methyl-4-isoxazol- propionic acid receptor (AMPAR) and the γ-amino-butyric acid B-receptor (GABAbR). It has been reported that anti-NMDAR encephalitis is the most common type of antibody-associated encephalitis. Although malignancies and infections have been considered triggers for anti-NMDAR encephalitis, environmental factors may represent a significant part of the risk in immune associated diseases.

Over the past few years, a series of studies have supported the role of the gut microbiota in the pathogenesis of neurologic diseases^3^. In 2012, Varrin-Doyer et al^4^. discovered that AQP4 p63–76 contains strong homology to aa 204–217 of an adenosine triphosphate-binding cassette (ABC) transporter permease of a gut microbe—*Clostridium perfringens*. This finding provides a possible connection between the gastrointestinal microbiota and molecular mimicry in the development of central nervous system (CNS) autoimmunity. Moreover, recent evidence suggest that the gut microbiota could modulate brain function and behaviors via the “microbiota-gut-brain” (MGB) axis^5^. For example, the gut microbiota has been reported to be associated with anxiety^6^, memory^7^, cognition^8^, and hyperactivity^9^. These findings highlight the novel possibility that disturbances of the gut microbiota or MGB axis may contribute to the onset of CNS autoimmunity and neuropsychiatric manifestations.

However, investigations of the MGB axis and autoimmunity for anti-NMDAR encephalitis are relatively rare. A recent clinical study demonstrated that anti-NMDAR encephalitis patients exhibit a substantial alteration in fecal microbiota composition relative to that of a small cohort of treatment-naïve patients^10^. Emerging animal studies have shown that mice devoid of gut microbiota from birth have reduced levels of NMDARs^11^, indicating possible connections between the gut microbiome and NMDARs. The past decade has yielded substantial evidence that the gut microbiome modulates brain function, including behaviors relevant to anxiety and depression^12^. In addition, ~80% of anti-NMDAR encephalitis patients develop pronounced psychiatric and behavioral symptoms and memory dysfunction^13^, including anxiety, irritability, auditory or visual hallucinations, sexual disinhibition, mania, cognitive disorder, and psychosis. These aforementioned studies suggest that gut microbiome dysbiosis may be associated with the development of anti-NMDAR encephalitis and neuropsychiatric manifestations. Meanwhile, the immune response mediated by gut microbiome was unknown in anti-NMDAR encephalitis.

To address this issue, a 16S ribosomal RNA (16S rRNA) gene sequencing-based approach was used to compare the gut microbial communities of patients with anti-NMDAR encephalitis and healthy controls (HCs) to evaluate whether gut microbiota dysbiosis was linked with anti-NMDAR encephalitis progress. To verify intestinal mucosal barrier dysfunction, levels of lipopolysaccharide (LPS), serum d-lactate (d-Lac) and diamine oxidase (DAO), markers of intestinal permeability damage, were also tested by enzyme-linked immunosorbent assay (ELISA). Then, we performed fecal microbiota transplantation (FMT) from anti-NMDAR encephalitis patients into microbiota-depleted mice to test whether anti-NMDAR encephalitis-relevant behavioral phenotypes were transmissible via their gut microbiota. Further investigation revealed that anti-NMDAR encephalitis FMT mice increased Th17 cells compared to HC FMT mice.

## Results

### Clinical characteristics of study participants

We studied the fecal microbiomes in a large and well-characterized cohort comprising 54 newly diagnosed subjects with anti-NMDAR encephalitis as well as 54 age-, gender- and body mass index (BMI)-matched HCs. Fourteen patients with antibiotic treatment or with missing data were excluded. Finally, 40 anti-NMDAR encephalitis patients and 54 HCs were included for further analysis. All the enrolled patients provided fecal samples before regular treatment. As shown in Table 1, the anti-NMDAR encephalitis patients (female: male = 22:18) were comparable to the HCs (female: male = 30:24), with a median age of 22.00 years among anti-NMDAR encephalitis patients and of 23.00 years among HCs. Among the 40 anti-NMDAR encephalitis patients, 32 patients presented with PCS (brief psychiatric rating scale (BPRS) >35), 6 patients with tumors and 15 patients with seizures.

**Table 1:** Demographic features of patients with anti-NMDAR encephalitis and HCs.

Demographic^a^ characteristic	Anti-NMDAR Encephalitis patients	Healthy controls	*P* value*
Sample size	40	54	–
Age (year)	22.00 (17.25–31.00)	23.00 (20.00–27.25)	0.3850
Gender (female:male)	22:18	30:24	–
BMI (kg m^−2^)	21.57 (18.86–24.88)	20.43 (19.39–22.08)	0.0802
Disease duration	28.23 ± 11.75		
*(d, mean* *±* *SD)*			
CSF anti-NMDAR Abs	40 (100%)		
positive (*n*, %)		–	–
With seizure (*n*, %)	15 (37.5%)		
With tumor (*n*, %)	6 (15%)	–	–
With PCS (*n*, %)	8 (%)	–	–
BPRS score	71.19 ± 10.02 (Non-PCS) 26.5 ± 3.34 (PCS)	–	–

BMI body mass index, PCS psychiatric symptoms, BPRS brief psychiatric rating scale.

^a^Mann–Whitney *U* test was used for continuous variables (age and BMI); values are expressed as the mean ± standard deviation if the data were normally distributed or as median and quartiles if the data were not normally distributed.

### Reduced alpha-diversity and altered overall microbial composition in anti-NMDAR encephalitis patients

**Fig. 1:**
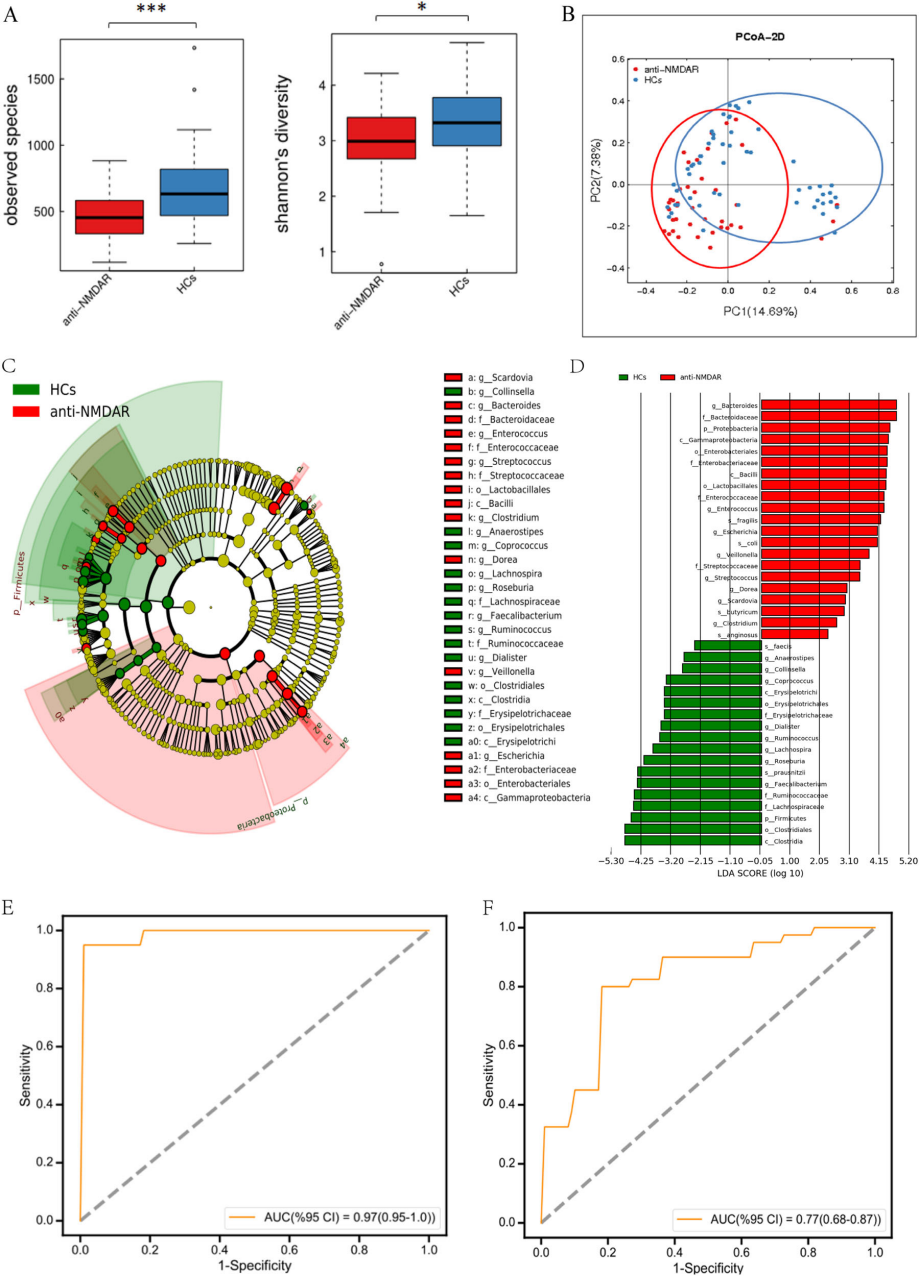
Gut microbial characteristics in anti-NMDAR encephalitis patients and HCs. **a** The number of observed OTUs and Shannon diversity index values were significantly reduced in anti-NMDAR encephalitis patients relative to the values in controls. **b** Principal coordinate analysis of Bray–Curtis dissimilarity demonstrated that individuals with anti-NMDAR encephalitis were significantly different from healthy controls (pseudo-F: 4.29, *p* < 0.001). **c** A cladogram of different taxonomic compositions in anti-NMDAR encephalitis patients (red) and healthy controls (green). **d** LDA scores showing significant bacterial differences between anti-NMDAR encephalitis patients (red) and healthy controls (green). Receiver operating characteristic analysis showed that the combination of **e** 50 microbial markers (including unidentified microbiome constituents) and **f** 10 identified genera can distinguish patients with anti-NMDAR encephalitis from HCs with an AUC of 0.97 and 0.77, respectively. (*p < 0.05, ***p < 0.001, by Mann–Whitney *U* test).

In total, we obtained 3913993 high-quality reads across all samples, which had an average length of 439.18 bp. These reads were clustered into 7096 operational taxonomic units (OTUs) at 97% sequence similarity with Greengene Database. Finally, 7096 qualified Operational Taxonomy Units (OTUs) were clustered for downstream analysis. A Venn diagram showed that 3536 of the 7096 OTUs were detected in the two groups, while 798 and 2738 OTUs were unique to patients with anti-NMDAR encephalitis and HCs, respectively (Fig. S1D). Alpha-diversity analysis showed that anti-NMDAR encephalitis was strongly associated with a decrease in intraindividual diversity, as measured by the Chao1, Observed Species, ACE, Shannon, and Simpson indexes (Figs. 1a and S1A, C).

To assess the overall diversity in gut microbiome composition, we performed principal coordinate analysis (PCoA) based on Bray–Curtis dissimilarity (pseudo-F: 4.29, *p* < 0.001, Fig. 1b). The microbial community between the two groups was also significantly different as measured by weighted UniFrac distance (pseudo-F: 7.36, *p* < 0.001, Fig. S1E) and unweighted UniFrac distance (pseudo-F: 2.88, *p* < 0.001, Fig. S1F).

### Differential distribution of intestinal microbiota bwtween anti-NMDAR encephalitis patients and HCs

To identify differentially abundant taxa, we performed linear discriminant analysis (LDA) effect size (LEfSe) analysis on the fecal microbiota composition of the two groups. *Bacteroidetes* and *Firmicutes* were the two most dominant phyla in both anti-NMDAR encephalitis patients and HCs (Figs. 1c and S2A). Moreover, at the phylum level, *Proteobacteria* was more abundant in anti-NMDAR encephalitis patients than in HCs, whereas the abundance of *Firmicutes* was higher in HCs (Fig. 1d). At the genus level, *Bacteroides*, *Prevotella*, *Faecalibacterium*, *Roseburia* and *Parabacteroides* dominated the gut microbiota in both groups (Fig. S2B). There were 31 bacterial taxa showing distinct relative abundances between the two groups (LDA score > 2.0, *p* < 0.05). The abundances of sixteen of these genera were significantly different. Decreased abundances of bacteria such as *Faecalibacterium*, *Roseburia*, *Lachnospira*, *Ruminococcus*, *Dialister*, *Coprococcus*, *Collinsella*, and *Anaerostipes* and increased abundance in *Bacteroides*, *Enterococcus*, *Escherichia*, *Veillonella*, *Streptococcus*, *Dorea*, *Scardovia* and *Clostridium* were observed observed in anti-NMDAR encephalitis patients relative to HCs.

### The gut microbiota distinguished anti-NMDAR encephalitis patients from healthy individuals

We next assessed the potential value of using the gut microbiota as biomarkers. A logistic regression analysis based on the relative abundance of different gut microbes was constructed, using 50 microbial markers in 40 patients and 54 controls (*P* < 0.01, Table S2). The receiver operating characteristic (ROC) analysis showed that this microbial panel, including unidentified microbiome constituents, enabled the discrimination of patients with anti-NMDAR encephalitis from HCs, with an area under the curve (AUC) of 0.97 (95% CI: 0.95–1.00) (Fig. 1e), confirming that the gut microbiome-based classifier is able to accurately distinguish anti-NMDAR encephalitis patients from controls. However, analysis of a separate cohort of patients would need to be analysed to test this model. Moreover, the ROC analysis of another microbial panel including 10 identified genera, *Acinetobacter*, *Anaerostipes*, *Anaerotruncus*, *Clostridium*, *Dialister*, *Enterococcus*, *Pseudoramibacter*, *Ruminococcus*, *Streptococcus* and *Veillonella*, yielded an AUC of only 0.77 (95% CI: 0.68–0.87) (Fig. 1f), suggesting the limitation of 16S rRNA gene sequencing (*P* < 0.01, Table S1).

### Microbial markers for different characteristics and diagnosis in anti-NMDAR encephalitis patients

**Fig. 2:**
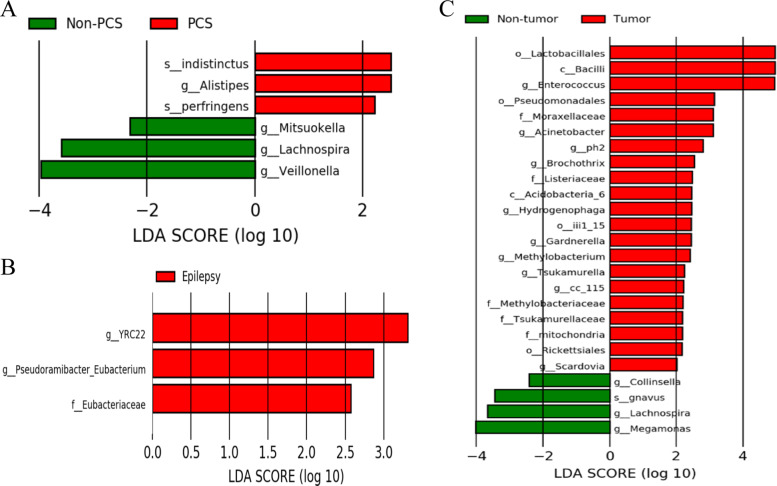
Differentially abundant microbes based on the LDA in anti-NMDAR encephalitis patients with different clinical characteristics. **a** LDA scores showing significant bacterial differences between the PCS subgroup (red) and non-PCS subgroup (green). **b** LDA scores showing significant bacterial differences between the epilepsy subgroup (red) and the non-epilepsy subgroup. **c** LDA scores showing significant bacterial differences between tumor subgroup (red) and non-tumor subgroup (green).

To determine whether disturbed microbiota in anti-NMDAR encephalitis patients might be linked with different clinical characteristics, we divided the patients into psychiatric symptoms (PCS) or non-PCS subgroups, epilepsy or non-epilepsy subgroups, and tumor or non-tumor subgroups. The abundances of the species *Latrodectus indistinctus* and *C. perfringens* and the genus *Alistipes* were higher in the PCS subgroup, whereas the genera *Mitsuokella*, *Lachnospira*, and *Veillonella* were more abundant in the non-PCS subgroup. (*P* < 0.05, LDA score > 2; Figs. 2a and S3A). Only the family *Eubacteriaceae* and the genera *YRC22* and *Pseudoramibacter* were more abundant in the epilepsy subgroup than in the non-epilepsy group (*P* < 0.05, LDA score > 2.5; Figs. 2b and S3B). There were 4 orders, 2 classes, 13 genera, 5 families and 1 species showing distinct relative abundances between the tumor and non-tumor subgroups (*P* < 0.05, LDA score >2; Figs. 2c and S3C).

### Association between fecal microbiome dysbiosis and increased intestinal mucosal permeability in anti-NMDAR encephalitis patients

**Fig. 3:**
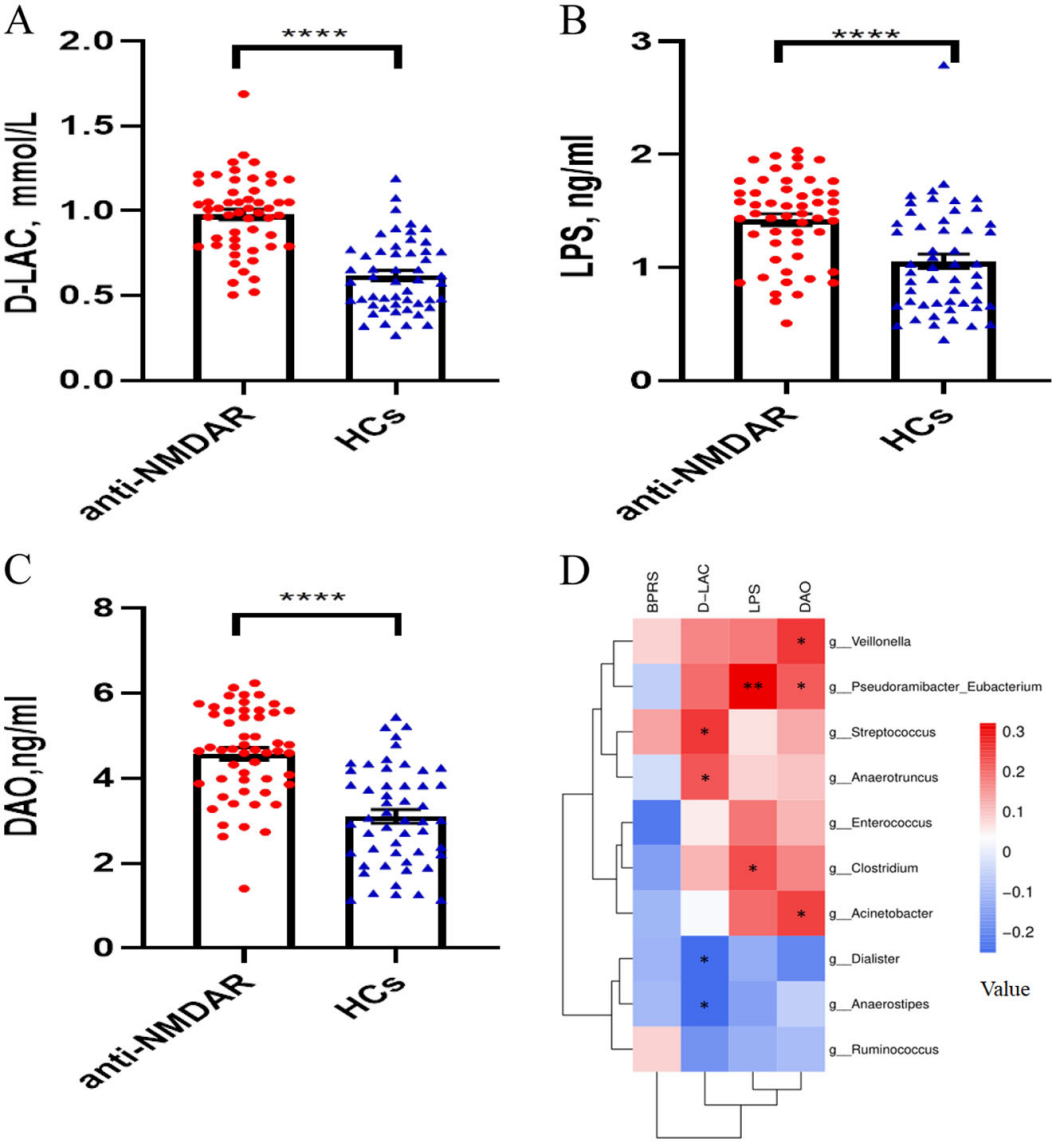
Concentrations of intestinal permeability damage markers and BPRS in anti-NMDAR encephalitis patients and HC, and their correlations with differentially abundant genera. **a** Serum D-Lac concentration in anti-NMDAR encephalitis and HC subjects. **b** Serum LPS concentration in anti-NMDAR encephalitis patients and HCs. **c** Serum D-Lac concentration in anti-NMDAR encephalitis patients and HCs. **d** Spearman’s rank correlation coefficient was calculated between the concentration of intestinal permeability damage markers, BPRS and the abundance of differentially abundant genera. (**a**–**c** ****p < 0.0001, by Mann–Whitney *U* test).

Because gut dysbiosis was observed in the microbiome of anti-NMDAR encephalitis patients, intestinal mucosal functions of anti-NMDAR encephalitis patients and HCs were determined. Pronounced increases in d-lac, DAO, and LPS were observed in anti-NMDAR encephalitis patients relative to the HC levels (*p* < 0.0001) (Fig. 3a, c). The average concentrations of d-Lac, DAO and LPS in anti-NMDAR encephalitis patients were 1.003 mmol/L, 4.632 ng/mL, and 1.415 ng/mL, respectively, while the average concentrations of D-Lac, DAO and LPS in HCs were 0.619 mmol/L, 3.112 ng/mL, and 1.071 ng/mL, respectively. When we further divided these patients into PCS and non-PCS subgroups, tumor and non-tumor subgroups, and epilepsy and non-epilepsy subgroups and performed subgroup comparisons, no significanct differences were found (Fig. S5). We also assessed the associations of intestinal mucosal functions and BPRS with the 10 differential genera (Fig. 3d). DAO was positively associated with *Acinetobacter*, *Pseudoramibacter* and *Veillonella* abundance, whereas LPS was positively associated with *Clostridium* and *Pseudoramibacter* abundance (*P* < 0.05). In addition, d-Lac was positively associated with *Streptococcus* and *Anaerotruncus* abundance but negatively correlated with *Dialister* and *Anaerostipes* abundance (*P* < 0.05). No significant associations were found between BPRS and the abundances of the differential genera.

### Microbial functional dysbiosis in anti-NMDAR encephalitis patients

**Fig. 4:**
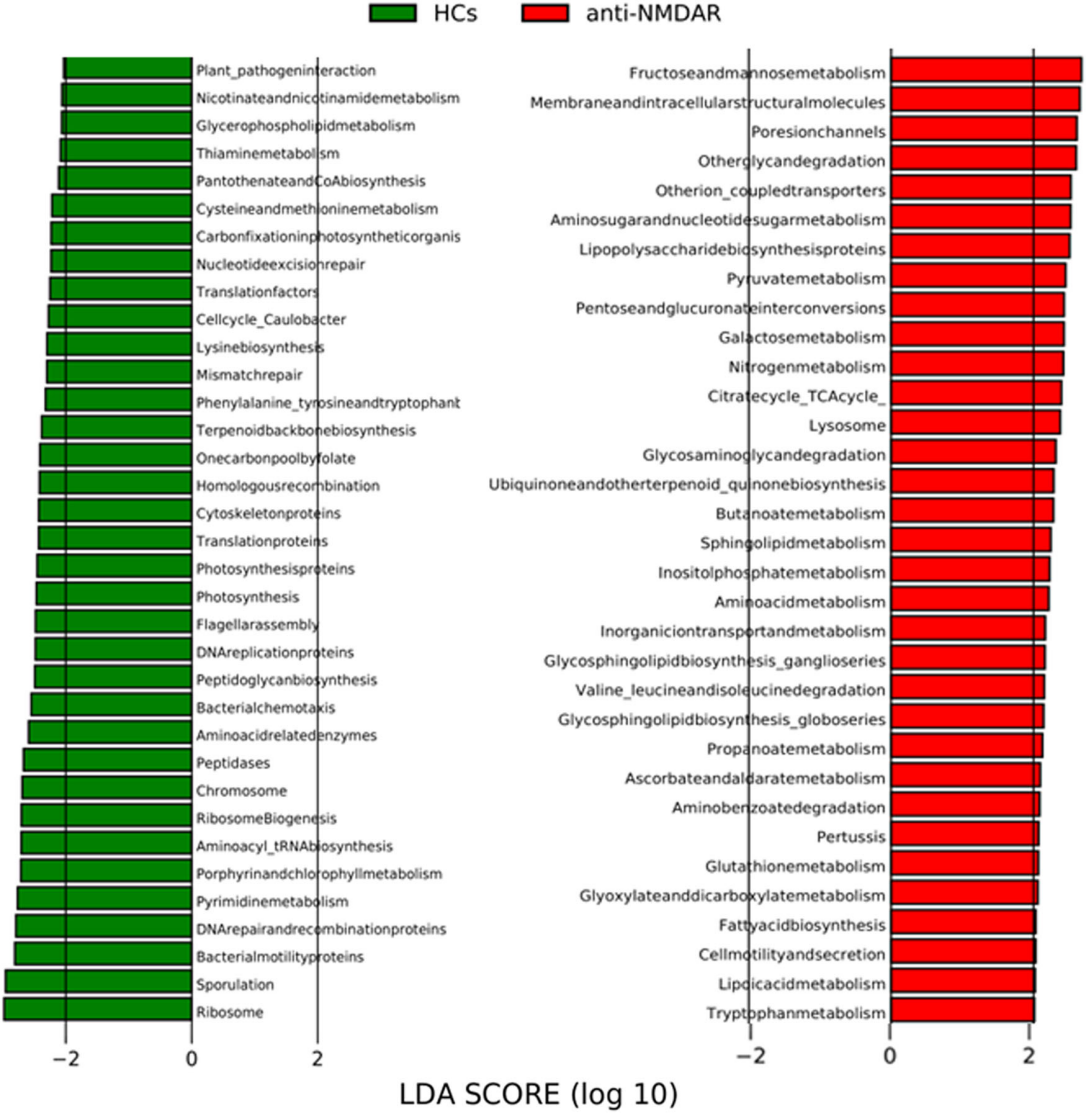
Functional analysis of predicted metagenomes, using PICRUSt. Bar plots of KEGG modules are significantly different between anti-NMDAR encephalitis patients and HCs.

To study the functional and metabolic changes of the microbial communities between anti-NMDAR encephalitis patients and HCs, we next inferred the metagenomes from the 16S rRNA data and analyzed the functional potential of the gut microbiota using Phylogenetic Investigation of Communities by Reconstruction of Unobserved States (PICRUSt). LEfSe analysis identified 68 Kyoto Encyclopedia of Genes and Genomes (KEGG) categories with significantly differential abundances between the anti-NMDAR encephalitis patients (*n* = 40) and HCs (*n* = 54) (LDA score > 2.0, *p* < 0.05) (Fig. 4). Of note, we found that functional modules related to fructose and mannose metabolism were significantly altered. In addition, LPS biosynthesis proteins appeared to be overrepresented in the microbiome of anti-NMDAR encephalitis patients relative to those in HCs, which is consistent with the increased LPS content in serum. The increased LPS biosynthesis, by inducing proinflammatory cytokines may allow antibodies access to the brain in anti-NMDAR encephalitis. Furthermore, multiple amino acid metabolisms were altered. For instance, tryptophan metabolism was increased in the microbiome of anti-NMDAR encephalitis patients. Glutathione metabolism was also highly enriched in the anti-NMDAR encephalitis microbiome.

### FMT from patients with anti-NMDAR encephalitis induces relevant behaviors in microbiota-depleted mice

**Fig. 5:**
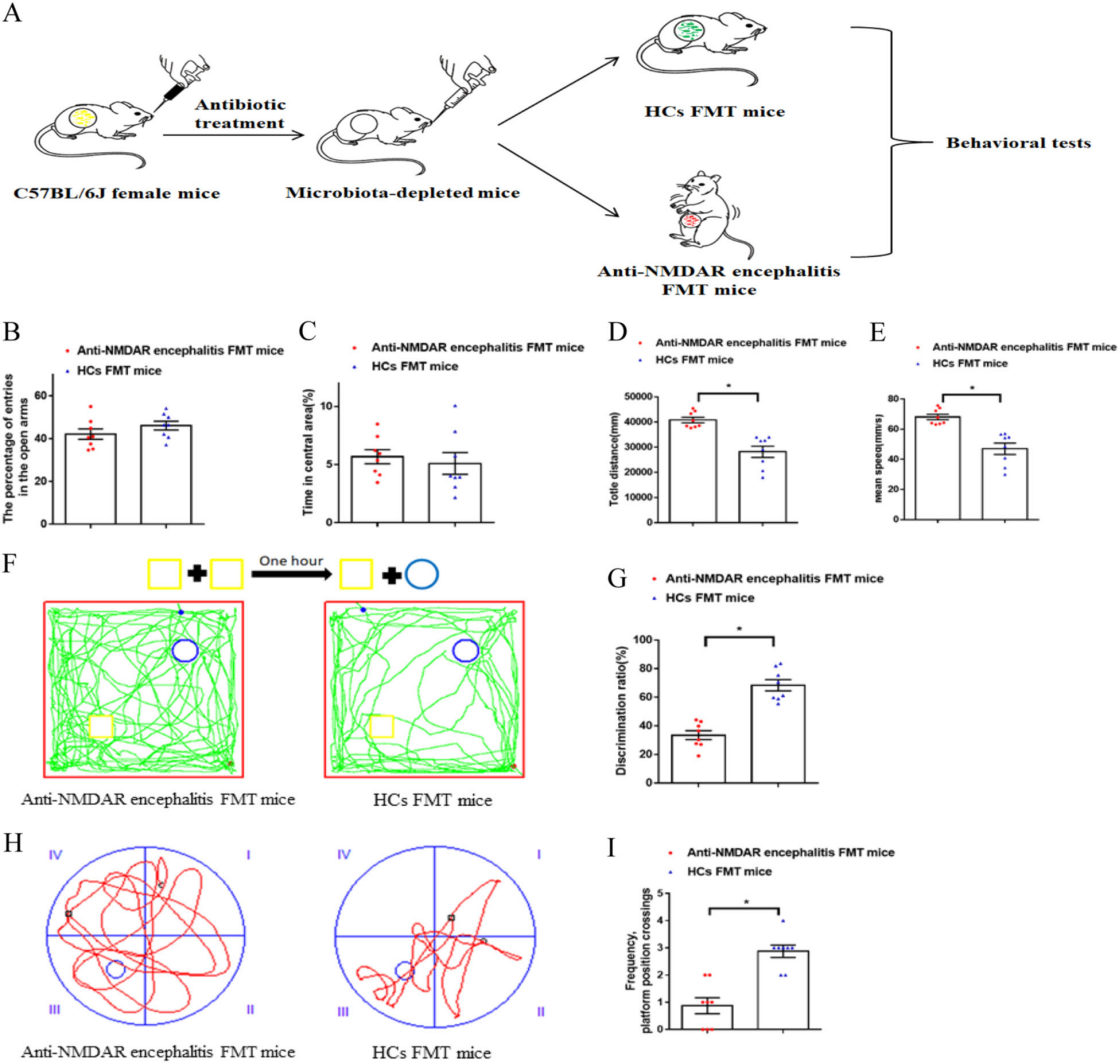
Behavioral comparisons between anti-NMDAR encephalitis FMT mice and HC FMT mice (*n* = 8). **a** The workflow diagram for this behavioral study. **b** Elevated plus maze test. No significant difference was observed between the HC FMT mice and anti-NMDAR encephalitis mice. **c**–**e** Open-field test. There was no significant difference between HC microbiota recipient mice and the anti-NMDAR encephalitis FMT mice in the time spent in the center area, but the total distance and mean total distance and speed were significantly increased in the anti-NMDAR encephalitis FMT mice. **f**, **g** Novel object recognition test. Relative to the HC FMT mice, the anti-NMDAR encephalitis FMT mice displayed a decreased discrimination ratio. **h**, **i** Morris water maze tests. Anti-NMDAR encephalitis FMT mice had significantly fewer platform position crossings than HC FMT mice. Each dot represents a mouse and experiments were repeated at least two times with similar results. (*P < 0.05, using nonparametric tests).

To determine whether disturbed gut microbiota in anti-NMDAR encephalitis patients might be linked with relevant behavioral phenotypes, we performed FMT experiments (Fig. 5a). The samples of the gut microbiota used for these FMT experiments were randomly selected. In an elevated plus maze test, the entries and time in the open arms did not differ between the anti-NMDAR encephalitis FMT mice and HC FMT mice, but distance and speed were significantly increased in the anti-NMDAR encephalitis FMT mice relative to those in the HC FMT mice (Figs. 5b and S4A, D), suggesting hyperactivity and the absence of depressive-like behavior. Similarly, the anti-NMDAR encephalitis microbiota recipient mice showed hyperactivity (increased total distance and mean speed; Fig. 5d, e) and no anxiety (no significance difference from HC mice in the time spent in the center area; Fig. 5c) in the open-field test (Fig. S4E). Cognitive behaviors were measured using Morris water maze tests and novel object recognition. Compared to the HC FMT mice, the anti-NMDAR encephalitis FMT mice displayed a lower discrimination ratio and lower discrimination index (Fig. S4G, H), higher speed and greater total distance (Fig. S4F, G) in the novel object recognition test (Fig. 5f), suggesting memory-learning deficits and hyperactivity. In the Morris water maze tests, the anti-NMDAR encephalitis FMT mice had significantly fewer platform position crossings than the HC FMT mice (Fig. 5h, i), indicating impaired spatial- and memory-learning deficits. During the test, anti-NMDAR encephalitis FMT mice also showed hyperactivity (increased distance and mean speed; Fig. S4I, J). Collectively, these four behavioral tests showed that anti-NMDAR encephalitis FMT mice displayed no anxiety-and depressive-like behaviors but hyperactivity and deficits in cognitive behavior, suggesting that the disturbed microbial composition of anti-NMDAR encephalitis FMT mice may be associated with behavioral characteristics in anti-NMDAR encephalitis patients.

### FMT from patients with anti-NMDAR encephalitis increases the proportion of IL-17-producing T Cells

**Fig. 6:**
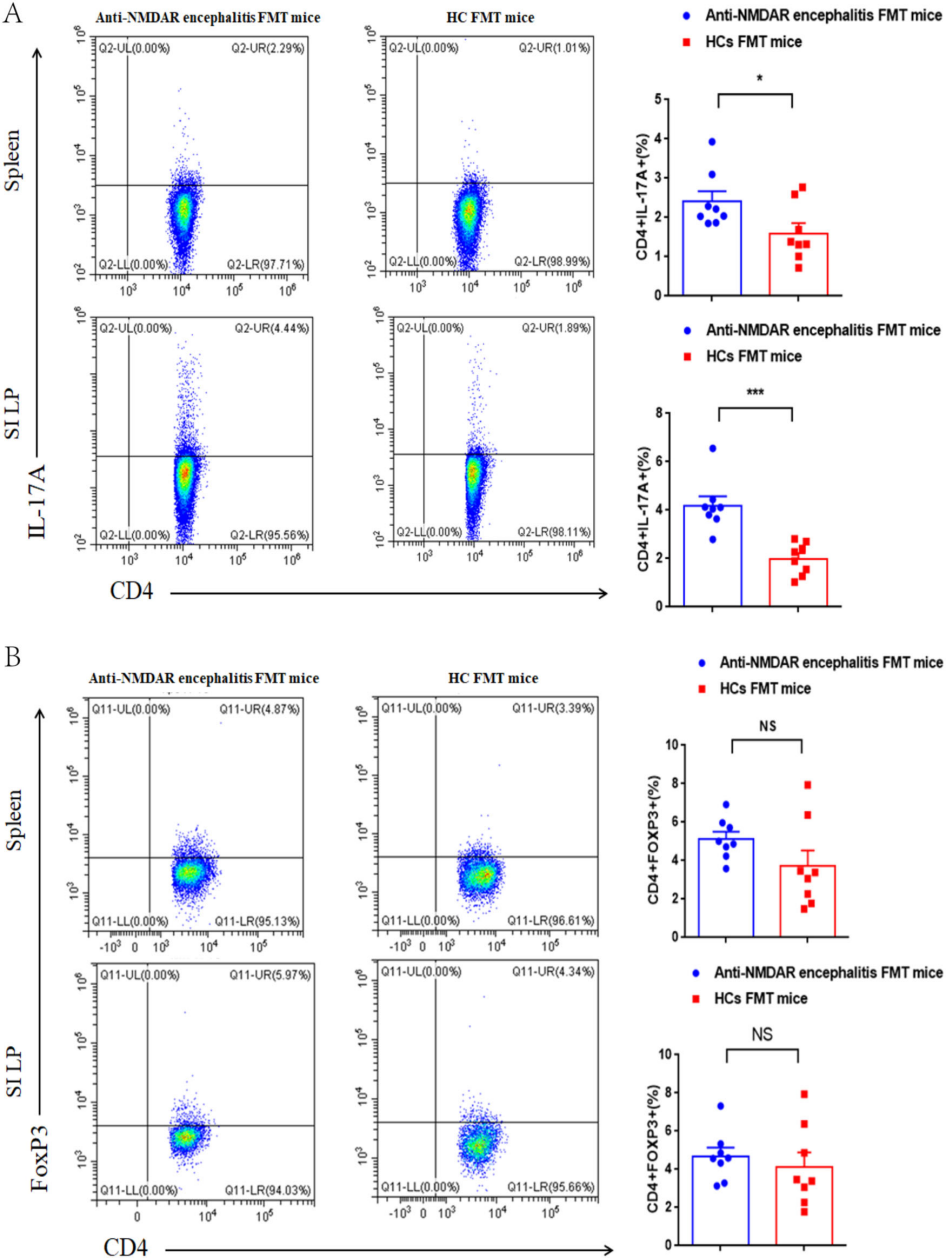
Increased Proportion of IL-17-Producing T Cells in the SI LP and spleen of anti-NMDAR encephalitis FMT mice (*n* = 8). **a** Representative staining of IL-17A^+^ CD4 T cell subsets and statistical analysis of the percentages in spleen and SI LP, gated on TCRβ + CD4^+^. **b** Representative staining of FOXP3^+^ CD4 T cell subsets and statistical analysis of the percentages in spleen and SI LP, gated on TCRβ + CD4^+^. Each dot represents a mouse and this is one of three different experiments performed with similar results in each experiment. (*P < 0.05, ***P < 0.001, using nonparametric tests).

Gut microbiota are believed to directly influence immune cells residing in the gut lamina propria^14^. IL-17-producing T cells and Tregs normally reside in the intestinal lamina propria^15^. To determine whether disturbed gut microbiota in anti-NMDAR encephalitis patients might be linked with immune response, lymphocytes from the spleen and small intestine lamina propria (SI LP) were isolated and the levels of Th17 and Treg cells were detected. Compared to the HC FMT mice, the anti-NMDAR encephalitis FMT mice displayed elevated proinflammatory Th17 cells (IL-17A^+^ CD4^+^ positive) in spleen and SI LP (Fig. 6a). However, no significant difference of Treg cells (Foxp^+^ CD4^+^ positive) was detected between the two groups (Fig. 6b). These results suggested that gut microbiota from anti-NMDAR encephalitis patients specifically enhanced Th17 response without affecting the percentage of Treg cells.

## Discussion

The gut microbiota can influence brain function, behaviors and neuroimmune modulation through the MGB axis and thus may predispose the onset of various neuroimmune disorders^16,17^. Herein, we first delineated the community structure of the fecal microbiota in a large and newly diagnosed anti-NMDAR encephalitis cohort by means of 16S rRNA gene sequencing. Our data demonstrate that anti-NMDAR encephalitis is characterized by a reduced bacterial diversity, altered relative abundances and intestinal mucosal barrier dysfunction. The most notable finding was that anti-NMDAR encephalitis-relevant behavioral phenotypes were transmissible via their gut microbiota. Moreover, gut microbiota itself can also modulate the immune responses through increasing Th17 cells.

Generally, high diversity is thought to be a marker of healthy status. Low bacterial diversity is recurrently documented in a variety of diseases and is considered to be one of the major types of gut dysbiosis^18^. As shown in the results, the fecal microbiome of patients with anti-NMDAR encephalitis exhibited decreased species richness and diversity compared with that of HCs. Moreover, the microbial composition in anti-NMDAR encephalitis patients was clearly different from that in HCs. The phylum *Firmicutes* was more abundant in HCs, while greater numbers of *Proteobacteria* were found in anti-NMDAR encephalitis patients, which is in accordance with our previous laboratory study in neuromyelitis optica spectrum disorders (NMOSDs)^19^. Notably, we observed a decrease in various short-chain fatty acid (SCFA)-producing bacteria, such as *Faecalibacterium*, *Roseburia*, *Lachnospira*, *Ruminococcus*, and *Coprococcus*. SCFAs, produced by the fermentation of dietary fibers, are known to exert beneficial effects on health through anti-inflammatory effects^20^. Decreased production of SCFAs by the microbiota increases disruption of the gut barrier^21^. In verifying the damage of the intestinal mucosa, large increases of d-Lac and DAO, chemical markers that are usually high in serum during the destruction of the intestinal barrier, were observed in anti-NMDAR encephalitis patients^22^. Therefore, the lack of protection from anti-inflammatory metabolites of beneficial bacteria and abnormal intestinal permeability also play key roles in the pathogenesis of anti-NMDAR encephalitis. Notably, the *Streptococcus* genus, which has previously been the taxon most strongly linked to NMOSDs, also demonstrated correlations with anti-NMDAR encephalitis and D-Lac^19^, suggesting that it is associated with damage to the intestinal mucosa.

Although NMDAR antibodies could be detected in cerebrospinal fluid (both sensitivity and specificity of 100%), it is less sensitive and specific to detect the antibodies in serum, in which the misdiagnosis rate is 13%^23^. In the present study, a model composed of 50 OTU markers, including unidentified microbiome constituents, was able to accurately distinguish anti-NMDAR encephalitis patients from HCs with high accuracy (AUC = 0.97). Moreover, the AUC of another microbial panel including a combination of 10 identified genera was only 0.77. The reason for the unidentified microbiome constituents may be the limitation of 16S rRNA gene sequencing; thus, metagenomic sequencing may improve the AUC and data interpretation in terms of identified species level and function analysis. Taken together, these results provide suggestive evidence for the potential clinical diagnostic value and lay the groundwork for further identification of “signature patterns” of defined gut microbes in anti-NMDAR encephalitis.

It is worth noting that gut dysbiosis in anti-NMDAR encephalitis may be associated with different clinical characteristics, such as seizures, psychiatric disorders, and tumors. Several studies have reported that these three clinical characteristics are associated with changes in the gastrointestinal microbiota^24,25^. We further investigated the associations between different clinical characteristics and the gut microbiota. Interestingly, the *Veillonella* genus was abundant in the non-PCS group, which implies that it was negatively correlated with psychiatric disorder. However, it was also abundant in anti-NMDAR encephalitis patients. Previous studies have observed significantly increased levels of *Veillonella* in autoimmune hepatitis and bipolar depression^25,26^. Some possible explanations need to be mentioned. On the one hand, *Veillonella* may prevent the occurrence of psychiatric disorder via secreting certain metabolic substances in specific conditions. On the other hand, a few samples of non-PCS subjects can be disturbed by a variety of factors. Nevertheless, the differential microbiome among different clinical characteristics needs to be validated with larger samples and animal experiments.

Of note, we found that fructose and mannose metabolism appear to be overrepresented in the microbiome of anti-NMDAR encephalitis patients relative to those in the microbiome of HCs, which may be related to the increased serum level of LPS and neuroinflammation in anti-NMDAR encephalitis patients^27^. Similarly, studies in mice have shown that glucose and fructose even at a moderate dose can enhance tumorigenesis^28^, which suggests that abnormal fructose metabolism may be involved in the development of tumors in anti-NMDAR encephalitis. In addition, the LPS biosynthesis pathway was also altered in the microbiome of anti-NMDAR encephalitis, which is in agreement with our finding that LPS content was increased in the serum of anti-NMDAR encephalitis patients. In addition to the distinctly increased fructose and mannose metabolism and LPS biosynthesis pathways, the metagenomes of anti-NMDAR encephalitis patients were characterized by alteration of tryptophan metabolism. Tryptophan is degraded to several indole derivatives by intestinal bacteria, and indoles act as ligands for the aryl hydrocarbon receptor (AhR), which promotes epithelial barrier function through secreting IL-22^29^. This alteration may play a key role in intestinal mucosal dysfunction.

We also demonstrated that the transfer of the fecal microbiota from patients with anti-NMDAR encephalitis to microbiota-depleted mice induced relevant behavioral phenotypes compared to those in HC FMT mice. Behavioral phenotypes seen in mouse models have relevance to multiple human psychiatric disorders. Notably, we discovered that anti-NMDAR encephalitis FMT mice were not anxiety-like but hyperactive relative to HC FMT mice in these four behavioral tests. A previous study indicated that the gut microbiota modulates walking speed and patterns of locomotion^30^, which is in agreement with our finding that the gut microbiota induced hyperactivity in anti-NMDAR encephalitis FMT mice. In addition, we observed significant impairments in learning and memory tasks, suggesting that disruption of the gut microbial community is associated with cognitive ability. Similar memory deficits were reported in healthy mice transplanted with microbiota from mice fed a high-fat diet^31^. Many factors have been suggested to mediate the relationship between gut microbial changes and the observed behavioral deficits. These factors may include increased gut permeability, elevated inflammation^31^, as observed in our study, and reduced synaptic plasticity^6,32^. Taken together, these results suggest that the gut microbiota may contribute to hypersensitivity and cognitive impairment in anti-NMDAR encephalitis, although these effects may not be disease specific.

Gut microbiota can modulate the immune response in a variety of ways, such as affecting antigen presentation and regulating the production of cytokines and the function of T lymphocytes^33^. We further investigated the effects of FMT on T cell responses which play an important role in regulation and prognosis of anti-NMDAR encephalitis immune damage^34^. The Th17 cells were also accumulated in the CSF of anti-NMDAR encephalitis patients than that of control individuals^34^. We found that the Th17 responses in the SI LP and spleen were increased in anti-NMDAR encephalitis FMT mice, while Treg response was not affected. The above mentioned gut microbiota alternation may be responsible for this effect. These results suggested the potential important involvement of gut microbiota effect on IL-17 in anti-NMDAR encephalitis. Further studies to explore the relationships of B cell and the gut microbes in anti-NMDAR encephalitis are highly wanted.

The major advantages of our study include the collection of newly diagnosed samples prior to treatment initiation. Nevertheless, several limitations in this study need to be noted. The gut microbiota can be influenced by multiple variables. First, host regional variation has a strong effect on gut microbiota composition^35^. The samples all come from South China and thus may limit the application of the microbiota-based diagnostic model. Second, the participants did not receive a standardized diet. The gut microbiota can be influenced by many diet compositions, such as dietary fats, dietary protein, dietary fiber. Dietary patterns including ketogenic diet, paleolithic diet, mediterranean diet, vegan/vegetarian diets, microbiota-targeted diets can also modulate microbial composition and metabolite production. The third limitation was the mechanistic explanation of the altered behaviour in animals. At last, we did not have a separate validation cohort of patients to test the biomarker model of exploration cohort as the small sample size. Longitudinal design with large cohort of patients before and after treatment is needed to clarify the diagnosis effect and cause-effect relationship between AIE and the gut microbiota.

In conclusion, this study suggests that the anti-NMDAR encephalitis microbiome itself can influence neurologic, Th17 response and behavioral function. The results suggested that an increase in immunogenic microbes and intestinal mucosal permeability and a decrease in anti-inflammatory metabolites are driving forces for the development and progression of anti-NMDAR encephalitis. Moreover, changes in the gut microbiota resulting from anti-NMDAR encephalitis FMT to mice led to hypersensitivity, cognitive impairment and increased Th17 cells in rodent models. Our findings provide a novel framework for understanding the mechanisms of anti-NMDAR encephalitis through the MGB axis and may lead to new therapeutic strategies.

## Materials and methods

### Study design

From October 2018 to May 2019, a total of 54 newly diagnosed subjects with anti-NMDAR encephalitis who were admitted to the Department of Neurology of the Third Affiliated Hospital of Sun Yat-sen University were enrolled in this study. Fourteen patients with antibiotic treatment or with missing data were excluded. Finally, 40 anti-NMDAR encephalitis patients (18 males and 22 females; median age, 22.00) and 54 HCs were included for further analysis. (Table 1) Diagnosis criteria for anti-NMDAR encephalitis were based on the diagnostic criteria by Graus et al.^36^. Cerebrospinal fluid (CSF) from patients was tested for IgG antibody against NMDAR by indirect immunostaining using a commercially available kit (EUROIMMUN Medizinische Labordiagnostika, Lübeck, Germany) according to the manufacturer’s instructions. When the patients were considered as anti-NMDAR encephalitis patients before treatment, we collected the stool samples first and then took the regular treatment in the meantime. A total of 54 healthy age-matched subjects (24 males and 30 females, median age, 23.00) (Table 1) were recruited from the outpatient setting as a control group. All the outpatients (control group) were recruited from the Medical Examination Center of the Third Affiliated Hospital of Sun Yat-sen University Hospital. The control group had to fulfill the following inclusion criteria^25^: (1) normal ranges of liver and kidney function tests, (2) normal fasting blood glucose, blood lipids, urine and stools, (3) did not take probiotics, prebiotics or antibiotics within 1 month before sample collection. The BPRS measured the severity of psychotic symptoms, ranged from 18 to 126, and was assessed by two senior psychiatrists. The control group was matched for BMI, age, and gender. This study was approved by the Medical Ethics Committee of the Third Affiliated Hospital of Sun Yat-sen University (approval ID: [2018]02-363-01). The subjects or the guardians of patients with severe cognitive impairment provided written informed consent for research and publication.

### Blood serum analysis

Blood sera were stored at −80 °C until d-Lac and DAO assays were performed. Serum D-Lac and DAO concentrations, markers of intestinal permeability damage, were tested using commercially available enzyme-linked immunosorbent assay kits (Cloud-Clone, Wuhan, China) according to the manufacturer’s instructions.

### Animal experiments and regents

Animal protocols were performed using C57BL/6J wild-type (WT) female mice in accordance with guidelines for animal care, according to the National Institutes of Health Guide for Care and Use of Laboratory Animals and approved by the Bioethics Committee of South China Agricultural University (approval ID: 2019-D063). C57BL/6J WT female mice were purchased from Guangdong Medical Laboratory Animal Center (Guangzhou, China). All mice were maintained under specific pathogen free conditions at South China Agricultural University (Guangzhou, China). Anti-CD4 FITC-conjugated, anti-Foxp3 PE-Cy7-conjugated, anti-IFNγ PE-Cy7-conjugated and anti-IL17A PE-conjugated antibodies were purchased from (Thermo Fisher Scientific, US).

### Fecal microbiota transplantation

After one week of acclimation, 6-week-old female mice were randomly assigned to one of two experimental groups: an anti-NMDAR encephalitis FMT group and an HC FMT group. The mean of each cage (each with 3–4 mice/cage and 2 cages/group) for all three independent replicate FMT experiments were analyzed, rather than the individual mouse. As described previously^37^, six-week-old C57BL/6 female mice were treated with a cocktail of antibiotics to deplete their gut flora and then used as recipients of FMT. The antibiotic treatment consisted of a mixture of metronidazole, vancomycin, neomycin and ampicillin which were administered in the drinking water for 1 week (antibiotics per 1 L of water: 1 g of metronidazole, 500 mg of vancomycin, 1 g of neomycin, 1 g of ampicillin). In addition, 200 mL of the antibiotic mix was administered by oral gavage every other day for another week. For transplantation, fecal matter from randomly selected subsets of anti-NMDAR encephalitis patients and HCs was administered by oral gavage daily for one week to colonize the recipient mice. Fresh stools from patients were collected and immediately positioned in standard sterile anaerobic collection tubes and stored at −80 °C. Then 100 mg stool was resuspended in 1 ml of sterile saline and the solution was vigorously mixed for 10 s using a benchtop vortex, before centrifugation at 800 g for 3 min. The supernatant was collected and delivered to the recipient mice via oral gavage (200 μL each recipient) within 10 min to prevent changes in bacterial composition. Finally, mice underwent behavioral testing and flow cytometry testing 2 weeks after FMT.

### Behavioral tests

#### Elevated plus maze test

Anxiety-related behavior was evaluated in the elevated plus maze test as previously described^38^.

### Open-field test

Autonomous behavior was measured in the open-field test as described previously^39^.

### Novel object recognition test

Nonspatial learning and memory were evaluated in the novel object recognition test as described previously^40^, with slight modifications.

### Morris water maze test (MWM)

Spatial learning and memory were evaluated in Morris water maze as described in our previous work^41^, with slight modifications.

### 16S rDNA PCR and sequencing analysis

Stool samples were collected and 16S rRNA sequencing was performed as described in previous work^33,42^. Fresh fecal samples were immediately frozen at −80 °C for subsequent analysis. The bacterial DNA was extracted from fecal samples with a QIAamp DNA Stool Mini Kit (Qiagen, Germany) according to the manufacturer’s instructions. We amplified the highly conserved V4 region of the bacterial 16S ribosomal RNA gene using polymerase chain reaction (PCR). The PCR products were purified with AmpureXP beads (AGENCOURT) to remove nonspecific products. The Qualified libraries were paired-end sequenced on a MiSeq system, with the sequencing strategy PE250 (PE251 + 8 + 8 + 251) or PE300 (PE301 + 8 + 8 + 301) (MiSeq Reagent Kit).

### Flow cytometry

The spleen and SI LP were removed from mice for the flow cytometry analysis. For intracellular cytokine staining, spleen and lymphocytes isolated from designated organs were stimulated, fixed and permeabilized, as previously described^42,43^, followed by fluorescent-conjugated intracellular cytokine antibody staining. Intra-nuclear Foxp3 was stained using the Foxp3 Staining Buffer Set (eBioscience, San Diego, CA, USA). Samples were detected by CytoFLEX and data were analyzed using CytoExpert (Beckman Coulter).

### Statistical analysis

Analysis of demographic and clinical data was conducted with GraphPad Prism 6.0 software, using the unpaired two-tailed Student’s *t*-test, Mann–Whitney U test or chi-square test, with statistical significance determined at an alpha of 0.05. Statistical analyses of sequencing data were performed using the R package (version 2.15.3) and indices tools such as Chao 1, Simpson, Shannon, ACE, Observed Species and LEfSe. LEfSe analysis was applied to identify taxa or pathways differentially abundant between patients and controls. This method uses the nonparametric factorial Kruskal-Wallis sum-rank test to detect features with significant differential abundance and then uses LDA to calculate the effect size of each feature. The operating characteristic curves (ROC) were constructed, and the AUC of each was calculated to assess the diagnostic performance of the model with the sklearn package. The metagenomes of the gut microbiome were imputed from 16S rRNA sequences with PICRUSt for predictive functional profiling (Phylogenetic Investigation of Communities by Reconstruction of Unobserved States)^44^. Independent fecal microbiota translational animal experiments were repeated for at least two times. Adjusted *p*-value < 0.05 was according to Benjamini–Hochberg procedure in microbiota data and behavioural tests^45^.

## Supplementary information

Concentrations of intestinal permeability damage markers in anti-NMDAR encephalitis patients with different clinical characteristics.

Supplementary Figure Legends

Gut microbial composition differences between patients with anti-NMDAR encephalitis and HCs.

Taxonomic summary of the gut microbiota of anti-NMDAR encephalitis patients and HCs at the (A) phylum level and (B) genus level.

Identification of differentially abundant microbes based on the LEfSe pipeline in anti-NMDAR encephalitis patients with different clinical characteristics.

Behavioral comparisons between anti-NMDAR encephalitis FMT mice and HC FMT mice (n=8).
