# Supplementary figures and images for: Fecal microbiota transplantation from patients with autoimmune encephalitis modulates Th17 response and relevant behaviors in mice
Source: Cell Death Discov. 2020 Aug 11;6:75. doi: 10.1038/s41420-020-00309-8 (PMC7419566; doi:10.1038/s41420-020-00309-8)

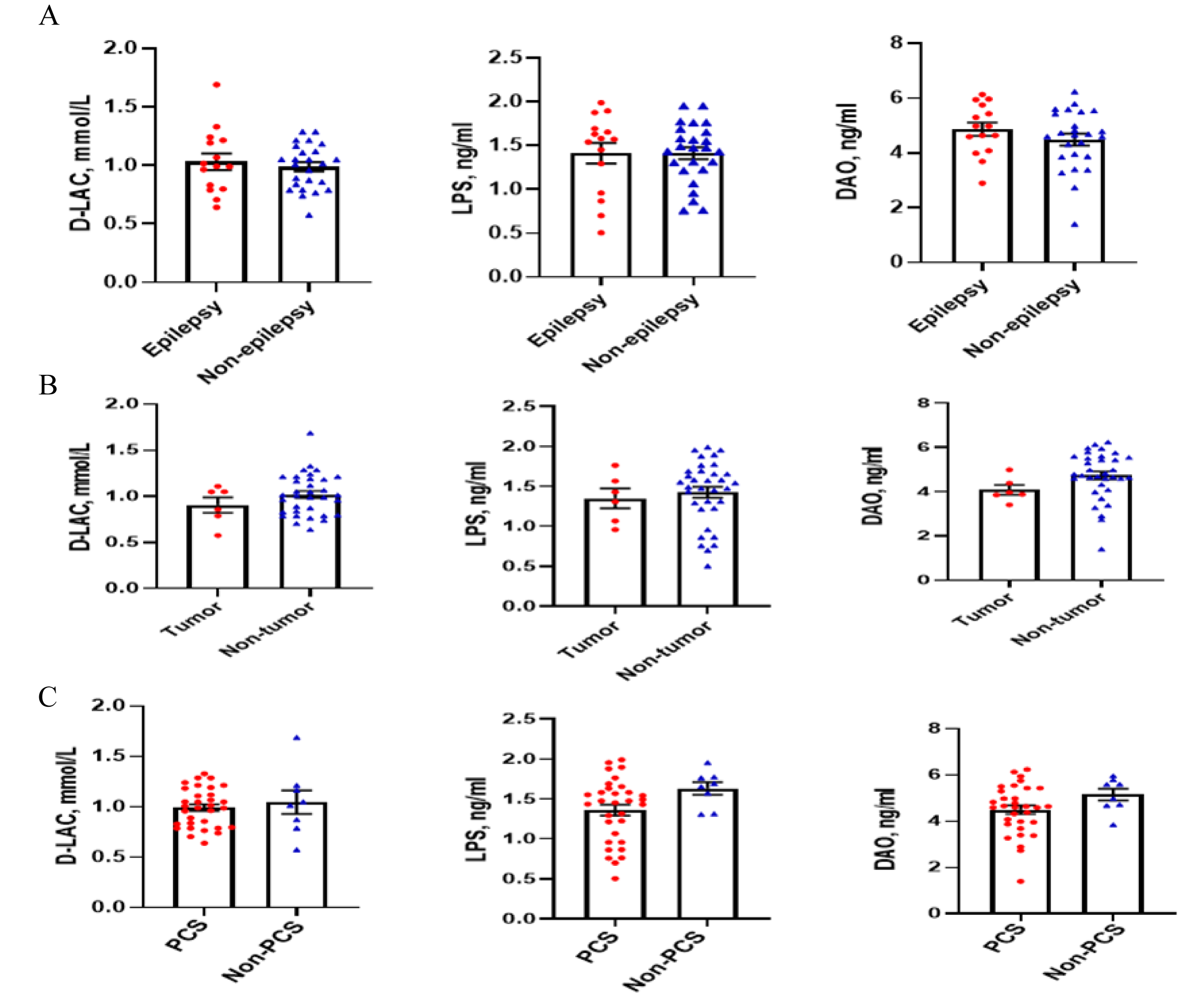

Supplement: Supplementary file 1 — Concentrations of intestinal permeability damage markers in anti-NMDAR encephalitis patients with different clinical characteristics. [file 41420_2020_309_MOESM1_ESM.tif]

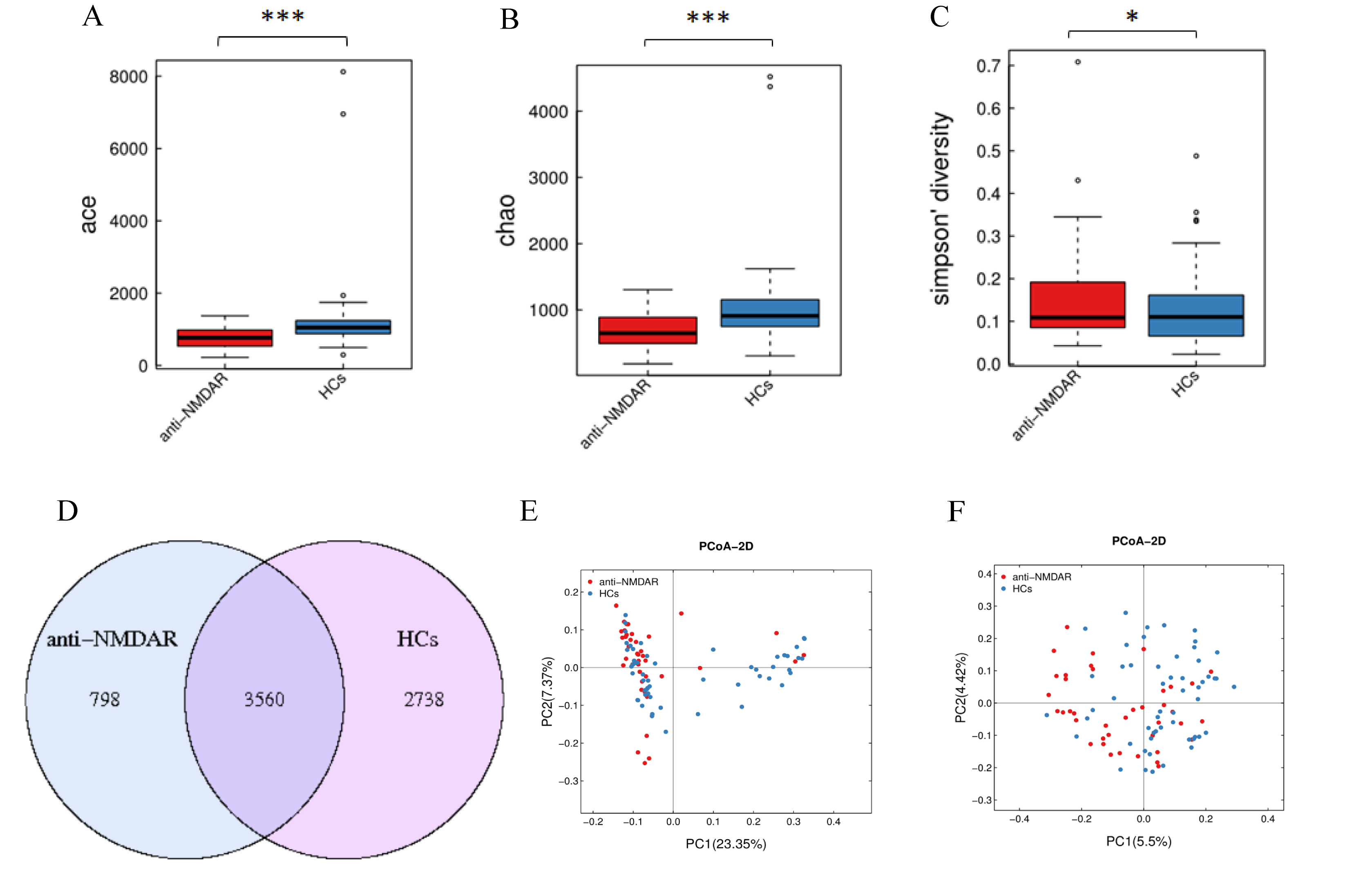

Supplement: Supplementary file 3 — Gut microbial composition differences between patients with anti-NMDAR encephalitis and HCs. [file 41420_2020_309_MOESM3_ESM.tif]

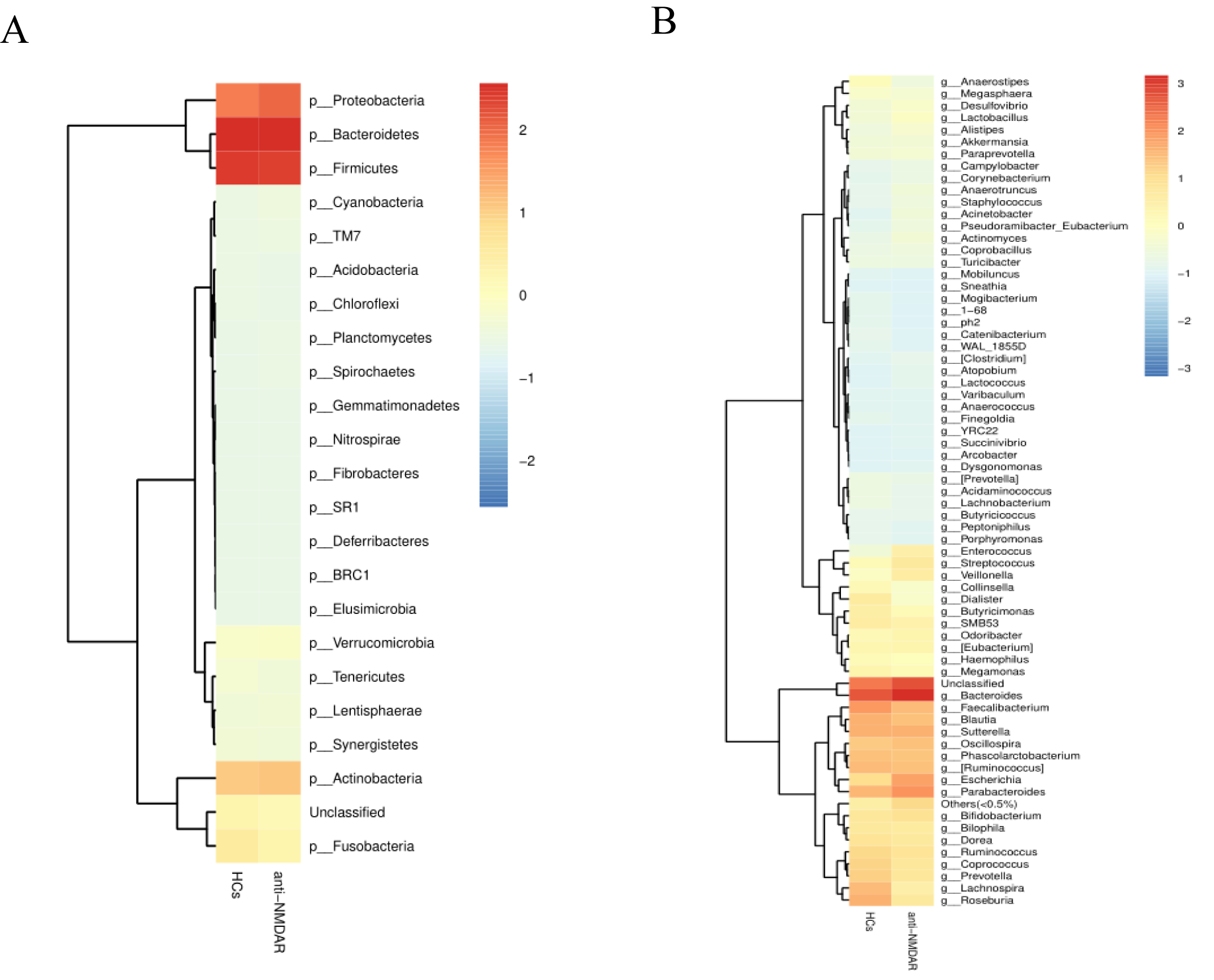

Supplement: Supplementary file 4 — Taxonomic summary of the gut microbiota of anti-NMDAR encephalitis patients and HCs at the (A) phylum level and (B) genus level. [file 41420_2020_309_MOESM4_ESM.tif]

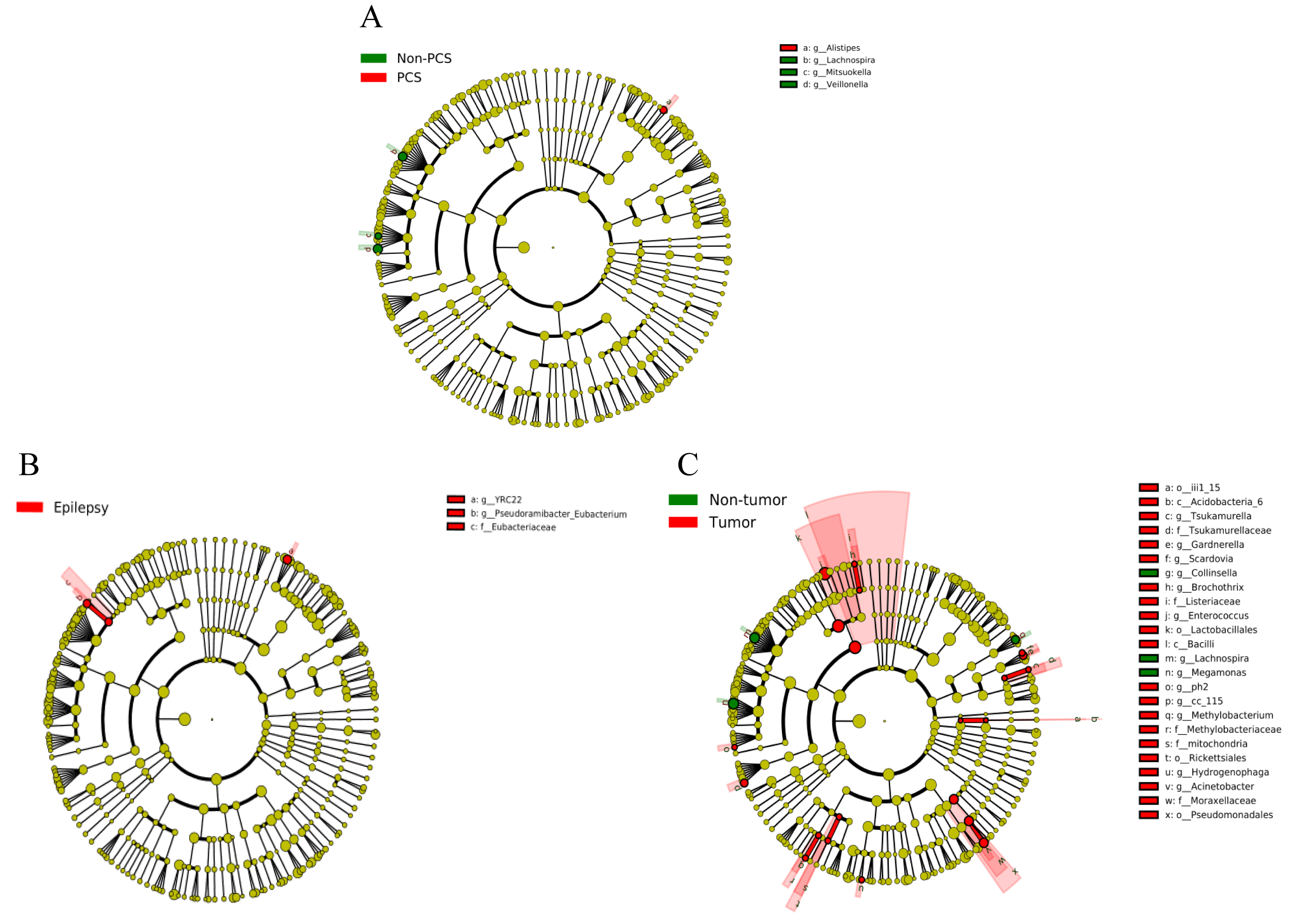

Supplement: Supplementary file 5 — Identification of differentially abundant microbes based on the LEfSe pipeline in anti-NMDAR encephalitis patients with different clinical characteristics. [file 41420_2020_309_MOESM5_ESM.tif]

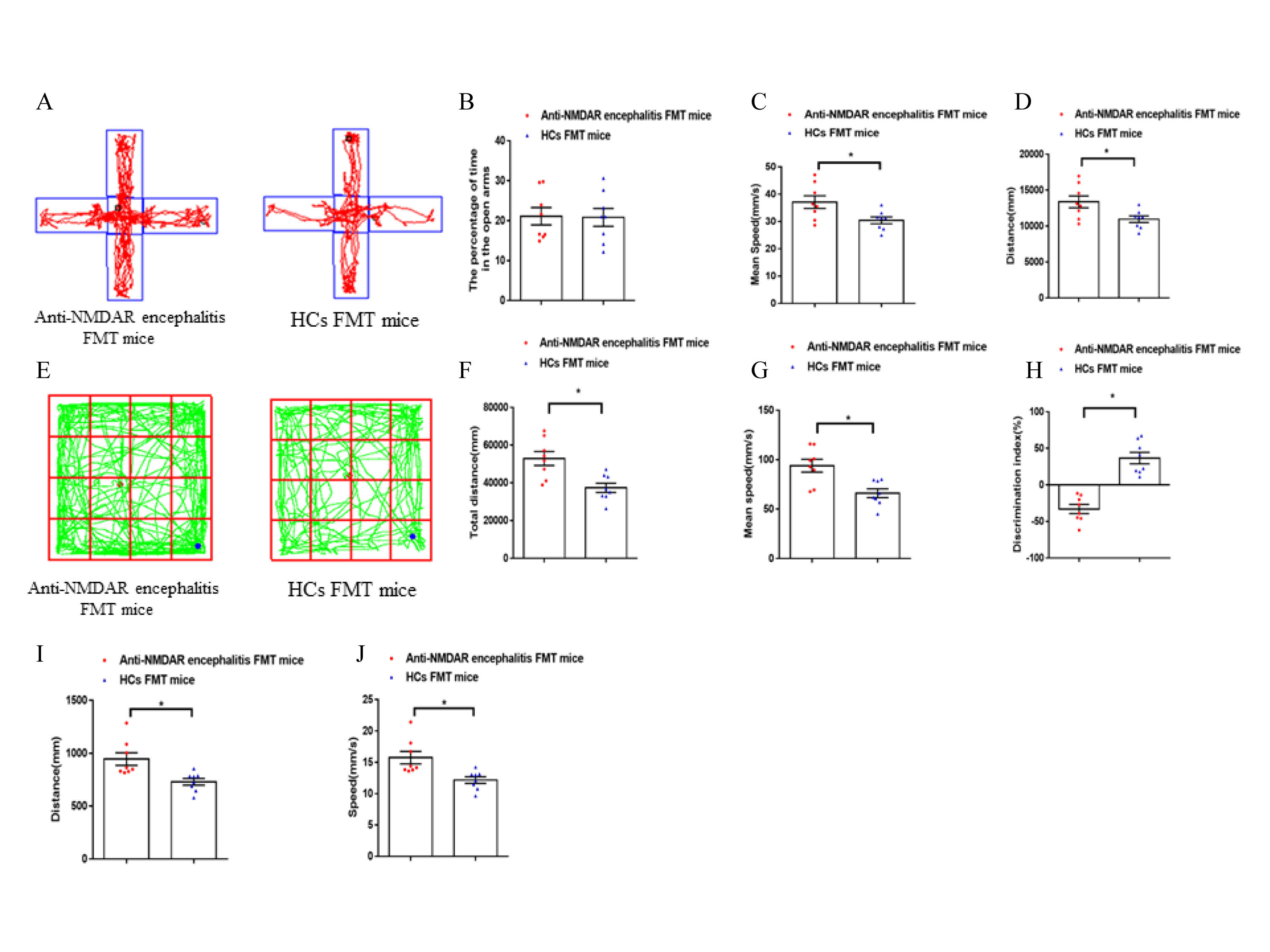

Supplement: Supplementary file 6 — Behavioral comparisons between anti-NMDAR encephalitis FMT mice and HC FMT mice (n=8). [file 41420_2020_309_MOESM6_ESM.tif]
