# Supplementary material for: Fecal microbiota transplantation from patients with autoimmune encephalitis modulates Th17 response and relevant behaviors in mice
Source: Cell Death Discov. 2020 Aug 11;6:75. doi: 10.1038/s41420-020-00309-8 (PMC7419566; doi:10.1038/s41420-020-00309-8)
Supplement: Supplementary file 2 — Supplementary Figure Legends [file 41420_2020_309_MOESM2_ESM.docx]

**Supplementary Materials**

**Fig. S1. Gut microbial composition differences between patients with anti-NMDAR encephalitis and HCs.** (**A-C**) Box plots depict greater gut microbial diversity in HCs than in anti-NMDAR encephalitis patients as measured by the ACE, Chao1, and Simpson indexes. (**D**) A Venn diagram showing that 3560 of 7096 OTUs were shared between the two groups, whereas 798 and 2738 OTUs were unique to patients with anti-NMDAR encephalitis and HCs, respectively. (**E**) PCoA of weighted UniFrac distance analysis demonstrating that individuals with anti-NMDAR encephalitis were significantly different from healthy controls (pseudo-F: 7.36, *p*<0.001). (**F**) PCoA of unweighted UniFrac distance analysis demonstrating that individuals with anti-NMDAR encephalitis were significantly different from healthy controls (pseudo-F: 2.88, *p*<0.001).

**Fig. S2. Taxonomic summary of the gut microbiota of anti-NMDAR encephalitis patients and HCs at the (A) phylum level and (B) genus level.**

**Fig. S3. Identification of differentially abundant microbes based on the LEfSe pipeline in anti-NMDAR encephalitis patients with different clinical characteristics.** (**A**) Cladogram based on the LEfSe method indicating the phylogenetic distribution of the intestinal microbiota associated with the non-PCS subgroup (green) and the PCS subgroup (red). (**B**) Cladogram based on the LEfSe method indicating the phylogenetic distribution of the intestinal microbiota associated with patients in the epilepsy subgroup (red). (**C**) Cladogram based on the LEfSe method indicating the phylogenetic distribution of the intestinal microbiota associated with patients in non-tumor subgroup (green) and the tumor subgroup (red).

**Fig. S4. Behavioral comparisons between anti-NMDAR encephalitis FMT mice and HC FMT mice (n=8).** (**A**). Representative paths in the elevated plus maze test. (**B-D**) Elevated plus maze test. The time in the open arms did not differ between the two groups, but distance and speed were significantly increased in the anti-NMDAR encephalitis FMT mice relative to the values in the HC FMT mice. (**E**) Representative paths of the open-field test. (**F-H**) Novel object recognition test. The anti-NMDAR encephalitis FMT mice displayed a decreased discrimination index high speed and decreased total distance relative to the values of the HC FMT mice. (**I and J**) Morris water maze tests. Anti-NMDAR encephalitis FMT mice showed greater distance and speed than the HC FMT mice.

**Fig. S5. Concentrations of intestinal permeability damage markers in anti-NMDAR encephalitis patients with different clinical characteristics.** (**A**) Concentrations of serum D-Lac, LPS, and DAO in the epilepsy subgroup and non-epilepsy subgroup. (**B**) Concentration of serum D-Lac, LPS, and DAO in the tumor subgroup and non-tumor subgroup. (**C**) Concentrations of serum D-Lac, LPS, DAO in the PCS subgroup and non-PCS subgroup.

**Table S1. Ten discriminatory differentially abundant genera between patients with anti- NMDAR encephalitis and HCs.**

|  | **Sample** | **Relative abundance of 10 differential genera** | | | | | | | | | |
| --- | --- | --- | --- | --- | --- | --- | --- | --- | --- | --- | --- |
|  |  | g__Acinet-obacter | g__Anaer-ostipes | g__Anaer-otru-ncus | g__Clostri-dium | g__Dialist-er | g__Entero-coccus | g__Pseudoram-ibacter_Eubact-erium | g__Rumin-ococcus | g__Strepto-coccus | g__Veillo-ella |
|  | HC1 | 0 | 0.023445 | 0 | 0.13188 | 1.403786 | 0 | 0 | 0.770764 | 0.011723 | 0.011723 |
|  | HC2 | 0 | 0.013085 | 0 | 0.268246 | 0.981386 | 0.003271 | 0 | 0.529949 | 0.17665 | 0.042527 |
|  | HC3 | 0 | 0.084167 | 0 | 0.12625 | 1.803114 | 0 | 0 | 2.359911 | 0.016186 | 0 |
|  | HC4 | 0 | 0.305169 | 0.022252 | 0.801068 | 0.27338 | 0.02861 | 0.003179 | 2.797381 | 0.08265 | 0 |
|  | HC5 | 0 | 0.015126 | 0.009076 | 0.323703 | 0.40841 | 0 | 0 | 0.980185 | 0.078657 | 0 |
|  | HC6 | 0 | 0 | 0.009517 | 0.418742 | 4.371411 | 0.003172 | 0 | 3.362624 | 0.275989 | 0.060273 |
|  | HC7 | 0 | 0.060469 | 0 | 0.108208 | 0.152764 | 0 | 0 | 2.35193 | 0.19732 | 0.114573 |
|  | HC8 | 0 | 0.030881 | 0 | 0.379841 | 3.0974 | 0 | 0 | 0.243963 | 0.126614 | 0.024705 |
|  | HC9 | 0 | 0.022381 | 0 | 0.089526 | 0 | 0 | 0 | 0.089526 | 0.006395 | 0.121499 |
|  | HC10 | 0 | 0 | 0 | 0.032108 | 0.429084 | 0.005838 | 0 | 0.280218 | 0.011676 | 0 |
|  | HC11 | 0 | 0.044423 | 0 | 0.190386 | 1.142313 | 0 | 0 | 1.577027 | 0.04125 | 0.13327 |
|  | HC12 | 0.002994 | 0 | 0 | 1.802179 | 0 | 0 | 0.005987 | 0.044905 | 0.008981 | 0.062867 |
|  | HC13 | 0 | 0.002763 | 0 | 0.038686 | 0.201719 | 0 | 0 | 0.013816 | 0.060792 | 0 |
|  | HC14 | 0 | 0.25799 | 0 | 0.151759 | 2.158011 | 0.00607 | 0 | 0.816463 | 0.154794 | 0.009106 |
|  | HC15 | 0.002964 | 0 | 0 | 1.007885 | 1.176854 | 0.038537 | 0 | 0.017786 | 0.14229 | 0 |
|  | HC16 | 0.003073 | 0.012291 | 0.003073 | 2.175516 | 2.362955 | 0.006146 | 0 | 0.009218 | 0.009218 | 0.009218 |
|  | HC17 | 0.003301 | 0.066028 | 0 | 1.033344 | 0 | 0 | 0 | 0.029713 | 0.009904 | 0 |
|  | HC18 | 0 | 0.047038 | 0 | 0.205792 | 0.029399 | 0 | 0 | 0.076437 | 0.044098 | 0.038218 |
|  | HC19 | 0 | 0.195346 | 0 | 0.045964 | 1.620224 | 0.005745 | 0 | 0.764148 | 0.0316 | 0.060327 |
|  | HC20 | 0 | 0.06876 | 0.003274 | 0.828395 | 2.603058 | 0.003274 | 0 | 3.359418 | 0.02292 | 0.029469 |
|  | HC21 | 0 | 0 | 0 | 1.009069 | 0.467391 | 0.009286 | 0 | 0.009286 | 0.040239 | 0.498344 |
|  | HC22 | 0.0031 | 0.034095 | 0 | 0.111583 | 0 | 0.012398 | 0 | 1.190218 | 0.043393 | 0.443232 |
|  | HC23 | 0 | 0.015305 | 0.012244 | 0.226507 | 0.015305 | 0.009183 | 0 | 1.068258 | 0.492807 | 0.247934 |
|  | HC24 | 0 | 0.002805 | 0 | 0.064522 | 3.453306 | 0 | 0 | 1.537296 | 0.030858 | 0.04769 |
|  | HC25 | 0 | 0.17691 | 0 | 0.425894 | 0.258813 | 0.003276 | 0 | 12.37387 | 0.006552 | 0.016381 |
|  | HC26 | 0 | 0.002762 | 0 | 0.011049 | 0 | 0.005525 | 0 | 0.168504 | 0.013812 | 0.008287 |
|  | HC27 | 0 | 0.018749 | 0 | 2.3936 | 0.028123 | 0.00625 | 0.003125 | 1.44366 | 0.015624 | 0.003125 |
|  | HC28 | 0.002964 | 0.044464 | 0 | 0.154143 | 0.083 | 0.005929 | 0 | 1.499926 | 0.083 | 0.002964 |
|  | HC29 | 0 | 0.128366 | 0 | 0.426891 | 0.152248 | 0.002985 | 0 | 0.217923 | 0.020897 | 0.005971 |
|  | HC30 | 0 | 0.110607 | 0.005821 | 0.04366 | 1.720224 | 0.011643 | 0 | 2.701129 | 0.017464 | 0.078589 |
|  | HC31 | 0 | 0.106691 | 0 | 0.731596 | 0 | 0.003048 | 0 | 0.533455 | 0.003048 | 0.01829 |
|  | HC32 | 0 | 0.014532 | 0 | 0.008719 | 5.429128 | 0.008719 | 0 | 0.014532 | 0.008719 | 0.014532 |
|  | HC33 | 0 | 0.040238 | 0 | 0.157856 | 2.541166 | 0.00619 | 0 | 0.578804 | 0.003095 | 0 |
|  | HC34 | 0 | 0.060149 | 0 | 0.712761 | 0.132327 | 0.003007 | 0 | 0.030074 | 0.003007 | 0.006015 |
|  | HC35 | 0 | 0.006415 | 0.006415 | 0.484347 | 0.022453 | 0.003208 | 0.009623 | 0.157172 | 0.022453 | 0 |
|  | HC36 | 0 | 0 | 0 | 0.239148 | 0 | 0.003276 | 0 | 1.808354 | 0.003276 | 0 |
|  | HC37 | 0 | 0.178316 | 0 | 0.057316 | 0 | 0.015921 | 0 | 2.321286 | 0.015921 | 0.006368 |
|  | HC38 | 0 | 0 | 0 | 0.076572 | 0 | 0.002836 | 0 | 0.011344 | 0.065228 | 0.062392 |
|  | HC39 | 0 | 0.132075 | 0 | 0.311321 | 0 | 0.009434 | 0 | 4.27044 | 0.018868 | 0.003145 |
|  | HC40 | 0 | 0.04961 | 0 | 0.180932 | 0.239297 | 0.002918 | 0 | 0.075875 | 0.002918 | 0.008755 |
|  | HC41 | 0.003205 | 2.730769 | 0.003205 | 0.435897 | 0.009615 | 0.003205 | 0 | 5.471154 | 0.009615 | 0 |
|  | HC42 | 0.070799 | 0.064362 | 0 | 0.936474 | 0.991182 | 0.016091 | 0 | 2.101435 | 0.099762 | 1.280814 |
|  | HC43 | 0 | 0.228522 | 0 | 0.966586 | 0.021617 | 0.018529 | 0 | 0.111173 | 0.04941 | 0.003088 |
|  | HC44 | 0 | 0.024227 | 0 | 0.043071 | 0.037687 | 0 | 0 | 0.228814 | 0.008076 | 0 |
|  | HC45 | 0 | 0.002737 | 0 | 0.013684 | 3.270478 | 0.002737 | 0 | 0.051999 | 0.005474 | 0.093051 |
|  | HC46 | 0.020937 | 0.19741 | 0 | 0.915263 | 0.472587 | 0.023928 | 0 | 0.753746 | 0.101696 | 0.463614 |
|  | HC47 | 0 | 0.027002 | 0 | 0.246017 | 0.003 | 0.012001 | 0 | 0.033002 | 0.051004 | 0.252018 |
|  | HC48 | 0 | 0.016768 | 0 | 0.041921 | 0.363311 | 0.027947 | 0 | 0.37449 | 0.033536 | 0.100609 |
|  | HC49 | 0 | 0 | 0 | 0.10142 | 0 | 0.002898 | 0 | 0.026079 | 0 | 0 |
|  | HC50 | 0 | 0 | 0 | 0.161735 | 0.285415 | 0.003171 | 0 | 0 | 0.22199 | 0.440808 |
|  | HC51 | 0 | 0.072186 | 0 | 1.092627 | 1.046691 | 0 | 0 | 0.836697 | 0.016406 | 0.075467 |
|  | HC52 | 0 | 0 | 0.003298 | 0.524458 | 0.15173 | 0.003298 | 0.029686 | 0.019791 | 0.019791 | 0.003298 |
|  | HC53 | 0.008891 | 0.029636 | 0.643096 | 1.466971 | 0 | 0.014818 | 0.017781 | 1.81371 | 0.062235 | 0 |
|  | HC54 | 0 | 0 | 0.057812 | 1.292094 | 0.008672 | 0.549212 | 0.361324 | 0.482729 | 0.020234 | 0 |
|  | Patient1 | 0 | 0.031115 | 0 | 0.043561 | 0.575625 | 0 | 0 | 0.479169 | 0.112013 | 0.031115 |
|  | Patient2 | 0 | 0.002824 | 0 | 0.07906 | 0.016941 | 1.027784 | 0 | 0.11012 | 1.296024 | 5.881522 |
|  | Patient3 | 0 | 0.064878 | 0 | 1.322953 | 0.282079 | 0.115653 | 0 | 1.794026 | 1.523229 | 0.062057 |
|  | Patient4 | 0 | 0.022093 | 0 | 0.093897 | 0.002762 | 0.008285 | 0 | 0.107705 | 0.171223 | 0.060757 |
|  | Patient5 | 0 | 0.134594 | 0 | 0.89933 | 0 | 0 | 0.006118 | 1.284757 | 0.048943 | 0.113181 |
|  | Patient6 | 0 | 0.002967 | 0 | 0.080104 | 0 | 0 | 0 | 0.020768 | 0.169109 | 0.005934 |
|  | Patient7 | 0 | 0.194296 | 0.012535 | 0.32905 | 0.877468 | 0.003134 | 0 | 2.817299 | 0.050141 | 0.043873 |
|  | Patient8 | 0 | 0 | 0 | 2.604843 | 0 | 0.028486 | 0 | 0.01266 | 1.221712 | 4.076594 |
|  | Patient9 | 0 | 0 | 0.016952 | 1.025598 | 0 | 0.002825 | 0.166695 | 0.6244 | 0 | 0.002825 |
|  | Patient10 | 1.418581 | 0 | 0.216926 | 0.082774 | 0 | 2.834309 | 0.07992 | 0.017126 | 0.005709 | 0.002854 |
|  | Patient11 | 0 | 0 | 0.145725 | 19.280297 | 0 | 13.130112 | 0 | 1.897398 | 0.104089 | 3.512268 |
|  | Patient12 | 0 | 0.008718 | 0.002906 | 0.450411 | 0.1482 | 0.002906 | 0 | 0.090082 | 0.034871 | 0.005812 |
|  | Patient13 | 0.015434 | 0 | 0.006174 | 2.321346 | 0 | 0 | 0.037043 | 0.101868 | 0 | 0 |
|  | Patient14 | 0 | 0 | 0 | 0.094116 | 0.002852 | 0.136896 | 0.102672 | 0.077004 | 0.037076 | 0.039928 |
|  | Patient15 | 0.005196 | 0 | 0 | 0.05196 | 0.010392 | 0.031176 | 0.033774 | 0.394898 | 0.376711 | 0.062352 |
|  | Patient16 | 0 | 0 | 0.006147 | 0.540956 | 0 | 0.301214 | 0.003074 | 0.018442 | 0.067619 | 0.245889 |
|  | Patient17 | 0 | 0 | 0.003144 | 0.069165 | 0 | 0 | 0 | 1.166373 | 0.119467 | 0.22007 |
|  | Patient18 | 0 | 0 | 0.272496 | 1.48261 | 0 | 0.00879 | 0.00293 | 0.01758 | 0.00586 | 0 |
|  | Patient19 | 0 | 0 | 0 | 0.356248 | 0.003019 | 0.084533 | 0 | 0.018114 | 0.220391 | 9.229236 |
|  | Patient20 | 0 | 0.018963 | 0 | 0.211302 | 0.530964 | 0.002709 | 0 | 0.295281 | 0.002709 | 0.005418 |
|  | Patient21 | 0 | 0.049677 | 0 | 2.449702 | 0.003105 | 0 | 0 | 0.7731 | 0.068306 | 0.145926 |
|  | Patient22 | 0.415387 | 0 | 0 | 0.020477 | 0.008776 | 15.948515 | 0 | 0.005851 | 0.014626 | 0 |
|  | Patient23 | 0 | 0.01841 | 0.003068 | 0.079777 | 0 | 0 | 0 | 0.33138 | 0.01841 | 0.01841 |
|  | Patient24 | 0 | 0 | 0.11567 | 1.056252 | 0 | 0.006088 | 0.018264 | 0.197857 | 0.045659 | 0.060879 |
|  | Patient25 | 0 | 0 | 0.035467 | 4.811728 | 0.005911 | 3.437371 | 0.254182 | 0.366495 | 0.082757 | 0 |
|  | Patient26 | 0.094824 | 0 | 0 | 0.002634 | 0.002634 | 83.798235 | 0 | 0.002634 | 1.772685 | 7.14869 |
|  | Patient27 | 0.009214 | 0 | 0 | 2.361794 | 0.003071 | 0.003071 | 0.015356 | 0.14742 | 0.07371 | 0.012285 |
|  | Patient28 | 0 | 0 | 0 | 0.006011 | 0.174321 | 0.015028 | 0 | 0.006011 | 6.200409 | 2.082832 |
|  | Patient29 | 0.009203 | 0.003068 | 0 | 1.941896 | 0 | 0.012271 | 0 | 0.113507 | 1.773169 | 3.356137 |
|  | Patient30 | 0.002969 | 0.002969 | 0 | 0.573057 | 0.068292 | 0.005938 | 0 | 0.056415 | 0.285044 | 0.041569 |
|  | Patient31 | 0 | 0 | 0.176185 | 0.870953 | 0 | 0.2194 | 0 | 0.069809 | 3.633402 | 0.003324 |
|  | Patient32 | 0.003113 | 0.133848 | 0.006225 | 0.547843 | 0 | 0 | 0 | 0.071593 | 0.006225 | 0.009338 |
|  | Patient33 | 0.015719 | 0.047158 | 0 | 1.097208 | 0.471579 | 0.003144 | 0.012575 | 0.08174 | 0.018863 | 13.855005 |
|  | Patient34 | 0.003038 | 0 | 0.057721 | 6.009053 | 0 | 0.182277 | 0.874928 | 0.288605 | 0 | 0.003038 |
|  | Patient35 | 1.354143 | 0 | 0.494734 | 0 | 0.00255 | 5.99036 | 0 | 0 | 0 | 0 |
|  | Patient36 | 0 | 0.031909 | 0 | 0.111682 | 0.041482 | 0.012764 | 0 | 1.123201 | 0.341428 | 0.296755 |
|  | Patient37 | 0.003157 | 0 | 0 | 0.220974 | 0 | 0.018941 | 0 | 0.00947 | 0.123114 | 0.034724 |
|  | Patient38 | 0.003194 | 0.012777 | 0.003194 | 0.137354 | 0.39609 | 0.038331 | 0.003194 | 1.0605 | 4.762665 | 1.651441 |
|  | Patient39 | 0.340267 | 0 | 0 | 0.025584 | 0.074193 | 44.247448 | 0.002558 | 0.007675 | 0.028142 | 0.012792 |
|  | Patient40 | 0.796945 | 0 | 0 | 0.309756 | 0.030073 | 0.947311 | 0.015037 | 0.036088 | 0.018044 | 0.009022 |

**Table S2. Fifty discriminatory OTUs between patients with anti-NMDAR encephalitis and HCs.**

| OTUs Taxonomic Assignment | |
| --- | --- |
| Otu1204 | k__Bacteria;p__Firmicutes;c__Clostridia;o__Clostridiales;f__Lachnospiraceae;g__Blautia;s__ |
| Otu2932 | k__Bacteria;p__Proteobacteria;c__Betaproteobacteria;o__Burkholderiales;f__Alcaligenaceae;g__Sutterella;s__ |
| Otu192 | k__Bacteria;p__Firmicutes;c__Clostridia;o__Clostridiales |
| Otu3325 | k__Bacteria;p__Firmicutes;c__Clostridia;o__Clostridiales;f__Ruminococcaceae;g__Faecalibacterium;s__prausnitzii |
| Otu2593 | k__Bacteria;p__Bacteroidetes;c__Bacteroidia;o__Bacteroidales;f__Prevotellaceae;g__Prevotella;s__stercorea |
| Otu3964 | k__Bacteria |
| Otu3968 | k__Bacteria;p__Firmicutes;c__Clostridia;o__Clostridiales;f__Lachnospiraceae;g__Blautia |
| Otu3570 | k__Bacteria;p__Bacteroidetes;c__Bacteroidia;o__Bacteroidales;f__Prevotellaceae;g__Prevotella |
| Otu618 | k__Bacteria;p__Firmicutes;c__Clostridia;o__Clostridiales;f__Ruminococcaceae;g__Faecalibacterium |
| Otu241 | k__Bacteria;p__Firmicutes;c__Clostridia;o__Clostridiales |
| Otu637 | k__Bacteria;p__Firmicutes;c__Clostridia;o__Clostridiales;f__Ruminococcaceae;g__Ruminococcus;s__ |
| Otu3738 | k__Bacteria;p__Firmicutes;c__Clostridia;o__Clostridiales;f__Lachnospiraceae;g__;s__ |
| Otu3228 | k__Bacteria;p__Bacteroidetes;c__Bacteroidia;o__Bacteroidales;f__Bacteroidaceae;g__Bacteroides |
| Otu3432 | k__Bacteria;p__Bacteroidetes;c__Bacteroidia;o__Bacteroidales;f__Bacteroidaceae;g__Bacteroides;s__ |
| Otu35 | k__Bacteria;p__Firmicutes;c__Clostridia;o__Clostridiales;f__Veillonellaceae;g__Dialister;s__ |
| Otu3445 | k__Bacteria;p__Bacteroidetes;c__Bacteroidia;o__Bacteroidales;f__Bacteroidaceae;g__Bacteroides |
| Otu3550 | k__Bacteria;p__Firmicutes;c__Clostridia;o__Clostridiales;f__Lachnospiraceae |
| Otu3889 | k__Bacteria;p__Bacteroidetes;c__Bacteroidia;o__Bacteroidales;f__Bacteroidaceae;g__Bacteroides |
| Otu3460 | k__Bacteria;p__Firmicutes;c__Clostridia;o__Clostridiales |
| Otu3862 | k__Bacteria;p__Bacteroidetes;c__Bacteroidia;o__Bacteroidales;f__Bacteroidaceae;g__Bacteroides |
| Otu2778 | k__Bacteria;p__Firmicutes;c__Clostridia;o__Clostridiales;f__Lachnospiraceae |
| Otu829 | k__Bacteria;p__Firmicutes;c__Clostridia;o__Clostridiales;f__Lachnospiraceae |
| Otu122 | k__Bacteria;p__Firmicutes;c__Clostridia;o__Clostridiales;f__[Tissierellaceae];g__Finegoldia;s__ |
| Otu2524 | k__Bacteria;p__Firmicutes;c__Clostridia;o__Clostridiales;f__Lachnospiraceae |
| Otu2526 | k__Bacteria;p__Firmicutes |
| Otu3802 | k__Bacteria;p__Firmicutes;c__Clostridia;o__Clostridiales;f__Lachnospiraceae |
| Otu162 | k__Bacteria;p__Firmicutes;c__Clostridia;o__Clostridiales;f__[Tissierellaceae];g__Anaerococcus;s__ |
| Otu2258 | k__Bacteria;p__Bacteroidetes;c__Bacteroidia;o__Bacteroidales;f__Bacteroidaceae;g__Bacteroides;s__ |
| Otu1255 | k__Bacteria;p__Firmicutes;c__Clostridia;o__Clostridiales;f__Lachnospiraceae |
| Otu1812 | k__Bacteria;p__Bacteroidetes;c__Bacteroidia;o__Bacteroidales;f__Prevotellaceae;g__Prevotella;s__stercorea |
| Otu88 | k__Bacteria;p__Firmicutes;c__Clostridia;o__Clostridiales;f__Ruminococcaceae |
| Otu1517 | k__Bacteria;p__Proteobacteria;c__Betaproteobacteria;o__Burkholderiales;f__Alcaligenaceae;g__Sutterella;s__ |
| Otu6068 | k__Bacteria;p__Firmicutes;c__Clostridia;o__Clostridiales;f__Veillonellaceae;g__Phascolarctobacterium;s__ |
| Otu1604 | k__Bacteria;p__Firmicutes;c__Clostridia;o__Clostridiales;f__Lachnospiraceae;g__;s__ |
| Otu3951 | k__Bacteria;p__Firmicutes;c__Clostridia;o__Clostridiales;f__Lachnospiraceae |
| Otu622 | k__Bacteria;p__Firmicutes;c__Clostridia;o__Clostridiales;f__Ruminococcaceae;g__;s__ |
| Otu453 | k__Bacteria;p__Firmicutes;c__Clostridia;o__Clostridiales;f__Lachnospiraceae |
| Otu3496 | k__Bacteria;p__Bacteroidetes;c__Bacteroidia;o__Bacteroidales;f__Prevotellaceae;g__Prevotella;s__copri |
| Otu3729 | k__Bacteria;p__Firmicutes;c__Clostridia;o__Clostridiales;f__Lachnospiraceae |
| Otu4119 | k__Bacteria;p__Firmicutes;c__Clostridia;o__Clostridiales;f__Lachnospiraceae |
| Otu973 | k__Bacteria;p__Bacteroidetes;c__Bacteroidia;o__Bacteroidales;f__Prevotellaceae;g__Prevotella;s__copri |
| Otu689 | k__Bacteria;p__Proteobacteria;c__Alphaproteobacteria;o__Caulobacterales;f__Caulobacteraceae;g__;s__ |
| Otu6443 | k__Bacteria;p__Bacteroidetes;c__Bacteroidia;o__Bacteroidales;f__Bacteroidaceae;g__Bacteroides;s__ |
| Otu3782 | k__Bacteria;p__Firmicutes;c__Clostridia;o__Clostridiales;f__Lachnospiraceae |
| Otu487 | k__Bacteria;p__Firmicutes;c__Clostridia;o__Clostridiales |
| Otu1472 | k__Bacteria;p__Firmicutes;c__Clostridia;o__Clostridiales;f__Lachnospiraceae |
| Otu2107 | k__Bacteria;p__Firmicutes;c__Clostridia;o__Clostridiales;f__Veillonellaceae;g__Phascolarctobacterium;s__ |
| Otu1457 | k__Bacteria;p__Firmicutes;c__Clostridia;o__Clostridiales;f__Ruminococcaceae;g__Oscillospira |
| Otu3838 | k__Bacteria;p__Firmicutes;c__Clostridia;o__Clostridiales;f__Lachnospiraceae;g__Blautia;s__ |
| Otu2268 | k__Bacteria;p__Bacteroidetes;c__Bacteroidia;o__Bacteroidales;f__[Odoribacteraceae];g__Butyricimonas;s__ |
